# PARP1 inhibition in naïve mouse embryonic stem cells induces viral mimicry

**DOI:** 10.1093/nar/gkag537

**Published:** 2026-06-03

**Authors:** Jianming Xu, Simon D Schwarz, Kapila Gunasekera, Roland Steinacher, Anna Kuśnierczyk, Michael O Hottiger, Primo Schär

**Affiliations:** Department of Biomedicine, University of Basel, Mattenstrasse 28, 4058 Basel, Switzerland; Department of Molecular Mechanisms of Disease, University of Zürich, Winterthurerstrasse 190, 8057 Zurich, Switzerland; Department of Biomedicine, University of Basel, Mattenstrasse 28, 4058 Basel, Switzerland; Department of Molecular Mechanisms of Disease, University of Zürich, Winterthurerstrasse 190, 8057 Zurich, Switzerland; Department of Chemistry, Biochemistry and Pharmaceutical Sciences, University of Bern, Freiestrasse 3, 3012 Bern, Switzerland; Department of Biomedicine, University of Basel, Mattenstrasse 28, 4058 Basel, Switzerland; Proteomics and Modomics Experimental Core Facility (PROMEC), Department of Clinical and Molecular Medicine, Norwegian University of Science and Technology (NTNU) and St. Olavs Hospital Central Staff, Trondheim 7491, Norway; RNA Mass Spectrometry Platform, Department of Chemistry, Biochemistry and Pharmaceutical Sciences, Freiestrasse 3, University of Bern, 3012 Bern, Switzerland; Department of Molecular Mechanisms of Disease, University of Zürich, Winterthurerstrasse 190, 8057 Zurich, Switzerland; Department of Biomedicine, University of Basel, Mattenstrasse 28, 4058 Basel, Switzerland

## Abstract

PARP1/2 inhibitors (PARPi) are effective in cancer therapy due to their synthetic lethality in cells with defects in DNA double-strand break repair (DSBR). Here, we show that DSBR-proficient, naïve pluripotent mouse embryonic stem cells (mESC) exhibit high sensitivity towards PARP1/2 inhibition by talazoparib and olaparib. This sensitivity results from a two-tiered response of mESC to PARPi, starting with the activation of DNA stress signalling via ATM and followed by a p53-controlled, TET-TDG-dependent transcriptional response, including the de-repression of endogenous retroviral elements (ERVs). The resulting accumulation of double-stranded RNAs then elicits hallmarks of viral mimicry, marked by induction of type I interferon and necroptosis responses, alongside caspase activation. Accordingly, depletion of p53, TET, or TDG confers PARPi resistance in mESC. These findings highlight active DNA demethylation as a critical mediator of PARPi sensitivity in mESC and provide mechanistic insight into how DNA stress drives ERV expression in cells with accessible chromatin.

## Graphical Abstract

**Graphical Abstract:**
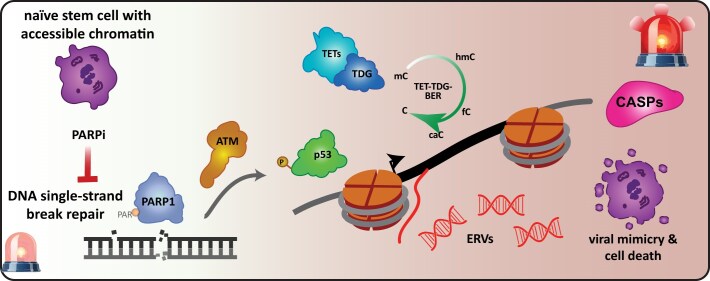

## Introduction

Protein ADP-ribosylation is catalysed by ADP-ribosyltransferases (ARTs). PARP1, the best-characterized ART family member, is responsible for the majority of nuclear ADP-ribosylation during genotoxic stress. Within the nucleus, post-translational ADP-ribosylation serves as a signal to organize multi-protein complexes associated with DNA damage response and repair [1], a function underlying the successful development of PARP1/2 inhibitors (PARPi) as anti-cancer drugs. PARPi in cancer therapy relies primarily on the concept of ‘synthetic lethality’, with cells carrying defects homologous recombination (HR, i.e. BRCA1 and BRCA2 deficiencies) failing to repair DNA double strand-breaks (DSBs) that accumulate following PARP1 inhibition [2, 3].

Mouse embryonic stem cells (mESC), derived from the inner cell mass of blastocysts, are pluripotent and can replicate indefinitely. When cultured in the presence of serum and leukaemia inhibitory factor (LIF), mESC assume a ‘primed’ state, in which the population is heterogeneous in terms of gene expression and developmental competence [4]. However, when cultured in ‘2i’ medium containing inhibitors of MEK1/2 and GSK3β, mESC remain in a naïve state of pluripotency, forming a more homogeneous population with high developmental competence [5]. Typically, genomes of 2i mESC are globally hypomethylated [6], a state that is established and maintained by both passive and active DNA demethylation [7, 8]. Active DNA demethylation involves dioxygenases of the ten-eleven translocation (TET) protein family oxidizing 5-methylcytosine (5mC) to 5-hydroxymethylcytosine (5hmC), 5-formylcytosine (5fC) and 5-carboxylcytosine (5caC) [9], and the Thymine DNA Glycosylase (TDG) to initiate the replacement of 5fC and 5caC with unmethylated cytosines via DNA base excision repair (BER), a process involving PARP1 [10–12].

Genomic regions that maintain DNA methylation under 2i conditions coincide with inactive chromatin and include transposable elements (TEs) and imprinted loci [13]. Among TEs are long terminal repeat (LTR)-containing endogenous retroviral elements (ERVs), which account for ~10% of the mouse genome [14]. While ERVs are stably silenced in most somatic cell types, predominantly by DNA methylation [15], their loci are more permissive for transcription in naïve mESC [16]. Low-level expression of certain ERVs in mESC and two-cell stage embryos has indeed been observed and was proposed to contribute to gene regulation [17]. The concept of ‘viral mimicry’ was introduced to describe a condition in somatic or cancer cells where the aberrant expression of ERVs resembles a viral infection and triggers an innate immune response [18–20]. Pluripotent embryonic stem cells have been reported to exhibit an intrinsically attenuated innate immune response to external viral stimuli [21–24]. This attenuation was proposed to serve as a protective mechanism in stem cells, preventing auto-reactivity against the spontaneous expression of ERVs within their hypomethylated genomes [25]. Despite this dampened response, mESC retain certain features of innate immunity, including the ability to respond to specific external stimuli [26]. However, it remains unclear whether and how these responses to exogenous challenges relate to the cell’s reaction to endogenous threats, such as the expression of ERVs.

Investigating the effects of PARP1/2 inhibition on viability of HR-competent cells, we found that naïve mESC showed high sensitivity. We identified p53, TET1/2, and TDG as central components of an underlying two-tiered PARPi response. A first-level response consists of genotoxic stress signalling via ATM and p53. A second level involves the p53-dependent activation of target genes, including ERVs, in a TET- and TDG-dependent manner. Altogether, we demonstrate a critical role for p53, TET-TDG-dependent active DNA demethylation, and ERV expression in activating a viral mimicry response, mediating cell death in PARP-inhibited, HR-proficient mESC.

## Materials and methods

### Inducible PARP1 deleted mESC

Parp1-floxed mice (*Parp1*^fl/fl^) were crossed with Rosa26::CreER mice [27] to generate PARP1 conditional KO mESC [28].

### Cell culture

Parp1^+/+^, *Parp1*^−/−^, and *Parp1*^fl/fl^ mouse ESCs were cultured in ‘2i’ medium (DMEM/F-12 and neurobasal medium supplemented with N2, B27, 0.1 mM 2-mercaptoethanol, 1× penicillin/streptomycin, 3 μM CHIR99021 GSK3β inhibitor, 1 μM PD0325901 MEK1/2 inhibitor, and 1000 U/ml LIF) on gelatinized plates. *Tdg*^+/+^, *Tdg*^−/−^, and *Tdg*^fl/fl^ mESC were cultured in ‘2i’ medium supplemented with 0.5 mM sodium pyruvate without antibiotics. For the transition into serum culture, 2i medium was replaced with Dulbecco’s modified Eagle’s medium (DMEM) GlutaMAX supplemented with 10% fetal bovine serum (FBS supreme, PAN Biotech), 10 mM sodium pyruvate, 0.1 mM 2-mercaptoethanol, 1× non-essential amino acids, 1× penicillin/streptomycin, and 1000 U/ml LIF. Cells were cultured for another five passages before adding Tal. The same medium was used to culture 159 mESC. NIH 3T3 cells were cultured in DMEM GlutaMAX supplemented with 10% FBS and 1× penicillin/streptomycin. HT-29 cells were cultured in DMEM GlutaMAX supplemented with 10% FBS. For small interfering RNA (siRNA) or double-stranded RNA (dsRNA) transfection, cells were reverse transfected with either Lipofectamine RNAiMAX or Mirus TransIT-X2 reagent. For treatments over multiple days, inhibitors were replenished in fresh medium every 24 h.

### Flow cytometry

For cell cycle analysis, mESC were treated as described and then harvested by trypsinization, followed by a single wash with ice-cold phosphate buffered saline (PBS). After washing, 100 μl cell suspension was held on ice until measurement. Directly before measurement the cell suspension was diluted with 900 μl DAPI staining solution (2 μg/ml DAPI, 50 mM Tris–HCl, pH 7.5, 150 mM NaCl, 2 mM MgCl_2_, and 0.1% Triton X-100) and analysed on a LSRFortessa (BD Biosciences) or a Cytoflex S (Beckman Coulter). Results were analysed with FlowJo, using mainly the Watson (pragmatic) model for cell cycle analysis, RMSD values were all <6.

### Immunofluorescence staining for γH2A.X

Cells grown on glass coverslips were treated as described and fixed with 4% formaldehyde. After fixation, cells were permeabilized with 0.2% Triton X-100 in PBS. Subsequently, cells were blocked by 2% bovine serum albumin (BSA) and 0.1% Triton X-100 in PBS. After blocking, cells were first stained with anti-γH2A.X Ab (Upstate, #05-636) followed by Cy3-conjugated goat anti-mouse secondary Ab (Jackson ImmunoResearch). Finally, cells were stained with Hoechst 33258 stain solution and mounted with Vectashield mounting solution.

### PFGE

Cells were treated as described and then harvested by trypsinization, followed by a single wash with cold PBS. 2.5 × 10^5^ cells in 100 μl PBS were mixed with an equal volume of pre-melted low melting point agarose. The mixture was transferred to the plug mold (BioRad) and plugs were subjected to proteinase K digestion in 5 ml lysis buffer [100 mM ethylenediaminetetraacetic acid (EDTA; pH 8.0), 0.2% sodium deoxycholate, 1% sarcosine, and 1 mg/ml proteinase K] at 37°C for 72 h. After digestion, the plug was washed three times with washing buffer (20 mM Tris–HCl, pH 8.0, and 50 mM EDTA) for 30 min each and subjected to electrophoresis for 21 h.

### RNA extraction, reverse transcription, and quantitative real-time PCR

Cells were treated as described and all cells cultured were harvested (including floating ones). Total RNA was extracted using either TRI Reagent (Sigma-Aldrich) or the RNeasy Plus Mini kit (QIAGEN). After extraction, 1 μg total RNA was digested with DNase I and reverse transcribed using either MultiScribe reverse transcriptase (ThermoFisher) or RevertAid First Strand cDNA synthesis kit (ThermoFisher) for first strand complementary DNA (cDNA) synthesis. Real-time PCR (polymerase chain reaction ) was performed with either the KAPA FAST SYBR kit (Roche) or the SensiFAST SYBR kit (Bioline) on Rotor-Gene Q (QIAGEN). Relative gene expression was determined using primers that spanned exon–exon junctions and either *Actb* or *Rps13* as an internal control for normalization.

### *In vitro* transcription and transfection

Sequences of the ERVs MLT1G, RMER19B, and RLTR45 were amplified from genomic DNA with primers containing a T7 promoter sequence. The resulting amplicons were *in vitro* transcribed using T7 RNA Pol (AmpliScribe) in the presence of RiboGuard RNAse inhibitor for 2 h at 37°C. Residual DNA was digested with DNAse I (ThermoFisher) and the RNA was purified using RNeasy plus Kit (Qiagen). Purity and integrity of the RNA were analysed with a Fragment Analyzer (Agilent). All mESC were transfected using 1 µg/ml dsRNA (or poly I:C, Tocris 4287) and the transfection agent TransIT-X2 (Mirus Bio) according to the manufacturer’s protocol.

### Library preparation and sequencing for RNA-seq

mRNA-seq: Total RNA was extracted with TRI Reagent. The quality of the isolated RNA was determined with a Qubit® (1.0) Fluorometer (Life Technologies) and a Bioanalyzer 2100 (Agilent). Only samples with a 260/280 nm ratio between 1.8 and 2.1 and a 28S/18S ratio within 1.5–2 were further processed. The TruSeq RNA Sample Prep Kit v4 (Illumina, Inc, California, USA) was used in the succeeding steps. Briefly, total RNA samples (100–1000 ng) were poly-A enriched and then reverse-transcribed into double-stranded cDNA. The cDNA samples were fragmented, end-repaired, and polyadenylated before the ligation of TruSeq adapters containing the index for multiplexing. Fragments containing TruSeq adapters on both ends were selectively enriched with PCR. The quality and quantity of the enriched libraries were validated using Qubit® (1.0) Fluorometer and the Caliper GX LabChip® GX (Caliper Life Sciences, Inc.). The product is a smear with an average fragment size of ~260 bp. The libraries were normalized to 10 nM in 10 mM Tris–Cl (pH 8.5) with 0.1% Tween 20. The TruSeq SR Cluster Kit HS3000 (Illumina) was used for cluster generation, using 10 pM of pooled, normalized libraries on the cBOT. Sequencing was performed on the Illumina HiSeq 4000 with single-end 150 bp using the TruSeq SBS Kit HS3000 (Illumina).

Total RNA-seq: Total RNA was extracted using TRI Reagent and subsequently ribo-depleted with the Illumina Ribo-Zero Plus rRNA Depletion Kit, following the manufacturer’s protocol. Libraries were sequenced using a single-end 100 bp approach to a depth of ∼90 million reads per sample.

### RNA-seq analysis

RNA-seq reads were aligned with the STAR-aligner [29]. As a reference, we used the Ensembl mouse genome build GRCm38. Gene expression values were computed with the function featureCounts from the R package Rsubread [30]. Differential expression was calculated using the generalized linear model implemented in the Bioconductor package DESeq2 [31]. The Sushi [32] framework was used to run the data analyses. Heatmaps were generated with R scripts using the function heatmap.2 of the R package gplots. In our RNA-seq analysis, genes that were identified as significantly differentially expressed by cuffdiff [33] were considered for further analysis after filtering for the genes with ≥2-fold increase/decrease in abundance.

### Genome-wide CRISPR screening

*Parp1* ^+/+^ mESC were first transduced with lentiviruses expressing Cas9 (lentiCas9-Blast) [34]. lentiCas9-Blast was a gift from Feng Zhang (Addgene viral prep #52962-LV; http://n2t.net/addgene:52962;  RRID: Addgene_52962). After selection with 10 μg/ml blasticidin, mESC stably expressing Cas9 were generated. For the screening, Cas9-expressing mESC were transduced with mouse Brie CRISPR knockout pooled lentiviral library [35], which was a gift from David Root and John Doench (Addgene #73633). One day after transduction, cells were selected with 0.75 μg/ml puromycin. Puromycin selection was kept until the cells were harvested. Four days after transduction, cells were treated with 5 nM talazoparib (Tal) for 4 days. Afterwards, the cells were grown without Tal for an additional 7 days to recover. Fifteen days after transduction, 5 nM Tal was added again. Cells were harvested at 14, 17, and 19 days after transduction. The library was prepared by amplifying the single guide RNA (sgRNA) sequences using Illumina Amplicon primers and sequenced on the MiSeq.

### Mass spectrometry of 5-methylcytosine and oxidative derivatives

DNA was extracted and purified with the Genomic Tip 100G Kit (Qiagen). High-performance liquid chromatography-tandem mass spectrometry analysis was performed on 10 µg genomic DNA digested to nucleosides in 10 mM ammonium acetate buffer (pH 6.0), 1 mM MgCl_2_ for 60 min at 40°C, with nuclease P1 from *Penicillium citrinum* (Sigma, N8630), Benzonase (Santa Cruz Biotech, sc-202391), and AP from *Escherichia coli* (Sigma P5931). Digested samples were precipitated with three volumes of acetonitrile and supernatants were lyophilized and dissolved in water for analysis. An Agilent 1290 Infinity II UHPLC system with an ZORBAX RRHD Eclipse Plus C18 150 × 2.1 mm ID (1.8 μm) column protected with an ZORBAX RRHD Eclipse Plus C18 5 × 2.1 mm ID (1.8 µm) guard column (Agilent) was used for chromatographic separation. The mobile phase consisted of A: water and B: methanol (both added 0.1% formic acid). Run started at 0.15 ml/min flow of 5% B for 3 min followed by 0.5 min gradient of 5%–13% B at 0.15–0.2 ml/min, 3 min of 13%–17% B at 0.2–0.25 ml/min, 1.5 min of 55%–80% B at 0.25 ml/min, 1.5 min of 80%–50% B at 0.25 ml/min, and 1 min of 50%–5% B at 0.25 ml/min, ending with 4 min re-equilibration with 5% B at 0.25 ml/min. Unmodified nucleosides were chromatographed isocratically with 20% B at 0.23 ml/min. An Agilent 6495 Triple Quadrupole system operating in positive electrospray ionization mode was used for spectrometric mass detection. The following mass transitions were monitored: 258.1/142.1 [5-hm(dC)]; 272.1/156.1 [5-ca(dC)]; 256.1/140.1 [5-f(dC)]; 252.1/136.1 (dA); 228.1/112.1 (dC); 268.1/152.1 (dG); and 243.1/127.1 (dT).

### SSB-seq and library preparation

The detection of endogenous single-strand breaks (SSBs) was performed as published before [36] but with considerable adaptations.

Briefly, genomic DNA was extracted with the Genomic-Tip 100/G Kit (Qiagen) from freshly treated mESC. Fifty micrograms gDNA was subjected to nick labelling using 5 μl *E. coli* DNA Polymerase I (NEB) and a labelling mix containing 2 μM DIG-dUTPs. The labelled DNA was purified and fragmented using a Bioruptor (Diagenode) and a restriction enzyme cocktail (EcoRI, HindIII, XbaI) to an average fragment size of 250 bp. Immunoprecipitation was performed overnight with 2 μg of anti-DIG Antibody (Roche) and the antibody-bound complexes were recovered with 40 μl preblocked Protein G Dynabeads (Invitrogen).

Libraries of input and IP samples (10 ng of DNA each) were prepared using the KAPA HyperPrep Kit (Roche) according to the manufacturer’s protocol. Subsequent paired-end sequencing (75 cycles) was performed on an Illumina MiSeq system at the genomics facility Basel to an average depth of ∼50 million reads per sample before filtering.

### SSB-seq analysis

Reads were aligned to the mouse genome (mm10 UCSC version) with bowtie2 (version 2.3.4.2) and extra options ‘--maxins 2000 --no-mixed --no-discordant --local --mm’. Duplicates were marked using Picard tools (version 2.9.2) and the resulting BAM files were filtered by removing reads that fell into ENCODE blacklist regions (version 2014, with manual removal of high-coverage artifact regions) using Rsamtools (R version 3.6, Bioconductor version 3.10), leading to an average coverage of at least 25 million reliable reads per sample.

Peaks were called across all replicates of a sample group using HOMER (version 2.1.2.1) with specifying commands ‘-style histone -o auto’. Genome-wide counts per million (CPM) were normalized to the total amount of filtered reads by applying a trimmed mean of M-values normalization using edgeR (Version 3.30). Analysis and visualizations were performed with the following packages under R version 4.0.3: GenomicRanges_1.14, ChIPpeakAnno_3.22.2, ChIPseeker_1.24, and metagene2_1.4, ggplot2_3.3.

Signals of SSBs outside of significant enrichment (peaks) were counted as log2 fold enrichment of IP over Input by using ‘bamCount’ (bamsignals package V1.22.0) and normalization by trimmed mean of M-values according to the library size using edgeR (V3.13).

### ATAC-seq, library preparation, sequencing, and processing

Tdg^fl/fl^ mESC were treated with 5 nM Tal for 24 h. According to the ‘Omni-ATAC’ seq protocol [37], potential cell-free DNA was digested with 200 U/ml DNase I in 2i medium for 30 min at 37°C. About 100 000 cells were harvested for each condition and replicate. Chromatin tagmentation and library preparation were done by using the ATAC-seq kit from Active Motif (#53150). ATAC-seq library was sequenced on the Illumina NovaSeq PE150 at Novogene (UK) to a coverage of at least 150 million reads per sample.

FastQC (v 0.11.9) was used to check the quality of the reads in ATACSeq fastq files and adaptor sequence contamination. Reads were mapped to the mm10 mouse genome assembly using STAR algorithm (v 2.7) with specifying commands --alignIntronMax 1 --outFilterScoreMinOverLread 0 --outFilterMatchNminOverLread 0 --outFilterMatchNmin 0 --outFilterMismatchNmax 2. High-resolution peak calling across replicates was performed using Genrich (v 0.6) with the following specifying commands: -p 0.005 -q 0.005 -a 20.0 -j -m 30 -r -v -l 50. Signals of SSBs or ATAC outside of significant enrichment (peaks) were counted as the log2 fold enrichment of IP over Input using ‘bamCount’ (bamsignals package, version 1.22.0) and normalized by the trimmed mean of M-values according to the library size, using edgeR (version 3.13).

### Protein extraction and analysis

Protein levels were analysed generally by western blotting of 50 µg NP-40 whole-cell extracts, separated by sodium dodecyl sulphate–polyacrylamide gel electrophoresis (SDS–PAGE) onto a nitrocellulose membrane (Amersham) and immunodetected with the following antibodies using a blocking solution with 5% non-fat dry milk in washing buffer (TBS with 0.1% Tween-20): anti-murine TDG Ab (L58, Lab P. Schär), anti-GAPDH Ab (Sigma-Aldrich, G9545; Santa Cruz, sc-25778), anti-β-Actin Ab (Sigma-Aldrich, A5441), anti-p53 Ab (Santa Cruz, sc-393031/sc-126), anti-Phospho-p53 (S15) Ab (Cell Signaling, #9284), anti-Phospho-p53 (S392) Ab (Santa Cruz, sc-51690), anti-53BP1 Ab (Santa Cruz, sc-22760), anti-PARP1 Ab (Santa Cruz, sc-7150/sc-53643), anti-Pol II Ab (Santa Cruz, sc-899), anti-Phospho-ATM (S1981) Ab (abcam, ab81292), anti-Chk1 Ab (Santa Cruz, sc-8408), anti-Phospho-Chk1 (S317) Ab (Cell Signaling, #2344), anti-PCNA Ab (Santa Cruz, sc-56), anti-Phospho-RPA32 (S33) Ab (Bethyl, A300-246A), anti-Histone H3 Ab (abcam, ab1791), anti-Phospho-H2A.X (S139) Ab (Biolegend, #613402), anti-Sox2 Ab (Santa Cruz, sc-365823), anti-Tubulin Ab (Sigma-Aldrich, T6199). Anti-phospho (Ser345) MLKL (D6E3G) Rabbit mAb, Cell Signaling #37333, was used on a PVDF membrane and blocking was done with TBS + 5% BSA.

### Chromatin immunoprecipitation (ChIP)

Cells were treated as described and crosslinked with 1% formaldehyde for 10 min at RT. Crosslinked cells were lysed in lysis buffer 1 (50 mM Hepes, pH 7.5, 140 mM NaCl, 1 mM EDTA, 10% glycerol, 0.5% NP-40, 0.25% Triton X-100, and 1× protease inhibitor cocktail). After centrifugation, the nuclear pellet was washed once with lysis buffer 2 (10 mM Tris–HCl, pH 8.0, 200 mM NaCl, 1 mM EDTA, 0.5 mM egtazic acid (EGTA), and 1× protease inhibitor cocktail) and lysed in lysis buffer 3 (10 mM Tris–HCl, pH 8.0, 140 mM NaCl, 1 mM EDTA, 0.5 mM EGTA, 0.5% sarcosine, 0.1% sodium deoxycholate, and 1× protease inhibitor cocktail). To shear the chromatin, the nuclear lysate was sonicated with a bioruptor and subject to immunoprecipitation with Ab coupled to Protein A/G Dynabeads in IP buffer (10 mM Tris–HCl, pH 8.0, 140 mM NaCl, 1 mM EDTA, 0.5 mM EGTA, 1% Triton X-100, 0.1% SDS, 0.1% sodium deoxycholate, and 1× protease inhibitor cocktail) overnight at 4°C. Beads were washed three times with high salt wash buffer (50 mM Hepes, pH 7.6, 0.5 M LiCl, 1 mM EDTA, 1% NP-40, 0.7% sodium deoxycholate, and 1× protease inhibitor cocktail) and subsequently once with low salt wash buffer (10 mM Tris–HCl, pH 8.0, 50 mM NaCl, and 1 mM EDTA). Immunoprecipitated complexes were eluted with prewarmed elution buffer (50 mM Tris–HCl, pH 8.0, 10 mM EDTA, and 1% SDS) and reverse crosslinked overnight at 65°C. Then, ChIP samples were subjected to RNase A digestion followed by Proteinase K digestion. DNA was extracted with NucleoSpin Gel and PCR Clean-up kit (Macherey-Nagel) and quantified by real-time PCR.

### Statistical analysis

To examine statistical differences, unpaired and two-tailed student’s *t*-tests were generally used to compare two groups of interest based on the variation over the biological replicates.

**Code:**

We relied on existing published analysis tools, as described in the ‘Materials and methods’ section. No new analysis tools were generated; scripts used to analyse the data using the published tools are available upon request.

## Results

### Naïve mESC are highly sensitive to PARPi

**Figure 1.**
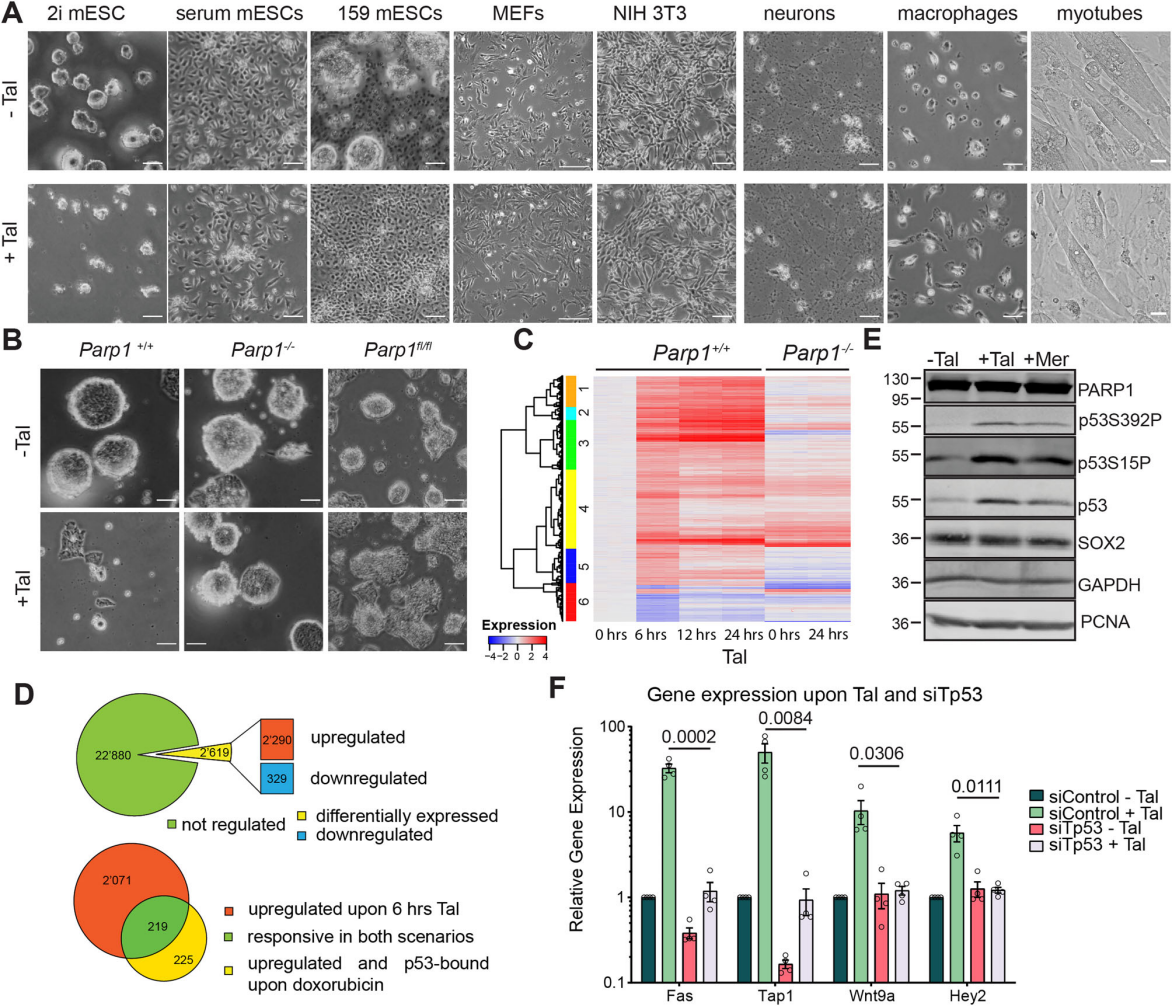
PARP1 inhibition is toxic in naïve mESC in a p53-dependent manner. (**A**) Representative depiction of the sensitivity of various cell lines to Tal treatment. Left: wild-type (wt) mESC (Ola129/E14) grown in 2i or serum, 159 mESC grown in 2i, and NIH 3T3 cells and MEFs treated with 5 nM Tal for 2 days. Scale bar: 50 µm. Right: post-mitotic neurons were first treated with 5 nM Tal for 5 days, followed by 50 nM Tal for an additional 4 days. Bone marrow-derived macrophages and differentiated C2C12 cells (myotubes) were treated with 50 nM Tal for 6 days. Scale bar: 20 µm. (**B**) Representative depictions of the sensitivity of mESC to Tal. *Parp1^+/+^, Parp1*^−/−^, and *Parp1^fl/fl^* (Cre-induction with 200 nM 4OHT, 3 days) mESC treated with 5 nM Tal for 2 days. Scale bar, 20 µm. (**C**) Heatmap with hierarchical clustering showing Z-scores of all differentially expressed genes in wt and *Parp1*^−/−^ mESC upon treatment with 5 nM Tal for periods indicated (*n* = 3). (**D**) Top: pie chart showing differential gene expression of wt mESC treated with 5 nM Tal for 6 h. Bottom: overlap between doxorubicin-induced p53 targets identified in mESC [44] and Tal-regulated genes. (**E**) Representative immunoblots showing proteins indicated in wt mESC treated with 5 nM Tal or 10 µM Mer for 6 h. (**F**) Relative mRNA expression by RT-qPCR in wt mESC treated with 5 nM Tal for 6 h upon p53 depletion (siRNA). Shown are means of relative expression + SEM (*n* = 3) versus dimethyl sulfoxide (DMSO) as control treatment. Numbers in graphs indicate *P*-values of unpaired, two-tailed *t*-tests between conditions.

Investigating the consequences of PARP1/2 inhibition in HR-competent cells, we subjected cells of different origins and types (primary, immortalized, proliferating, or post-mitotic) from mice to a low-dose (5 nM) Tal treatment [38]. As predicted from current models of PARPi action, HR-proficient NIH/3T3 cells, mouse embryonic fibroblast (MEFs), differentiated C2C12 cells (myotubes), bone marrow-derived macrophages, and post-mitotic neurons were all resistant to Tal treatment (Fig. 1A). This is consistent with previous findings showing that differentiated somatic cells exhibit minimal sensitivity to PARP inhibitors at such low concentrations, especially in the absence of exogenously induced DNA damage (e.g. MMS treatment) [2, 39]. In contrast, we observed naïve mESC (Ola129/E14) cultivated in 2i medium to be sensitive to Tal treatment (Fig. 1A). An unrelated mESC line (159), which tends to differentiate spontaneously in serum-containing media was similarly sensitive to Tal, but cell death was only apparent in the pluripotent cell fraction (spheres) and less so in the more differentiated cells (grown as monolayer) (Fig. 1A). Notably, mESC with either a constitutive (*Parp1*^−/−^) or a conditional PARP1 defect (*Parp1*^fl/fl^) were resistant to the treatment (Fig. 1B and Supplementary Fig. S1A), demonstrating that the observed toxicity of Tal depends on PARP1 activity. These effects were reproducible with olaparib, an alternative PARPi [40] (Supplementary Fig. S1B).

To further investigate the role of the pluripotency state in PARPi sensitivity, we conditioned ‘2i’ mESC in ‘serum + LIF’ to prime for differentiation. Treatment with Tal in serum + LIF resulted in a slight, statistically non-significant, reduction in cell viability (Supplementary Fig. S1C). Notably, this experiment also demonstrated that PARPi-mediated toxicity is independent of cell doubling speed: mESC in serum + LIF (and MEFs) showed reduced or no sensitivity to Tal but higher proliferation when compared to 2i conditions. Similar differences in duplication rates of stem cells across pluripotency states were reported previously [41] (Supplementary Fig. S1D). Moreover, the addition of ‘2i’ inhibitors did not sensitize NIH 3T3 cells to Tal (Supplementary Fig. S1E), indicating that the naïve pluripotent state, not the inhibition of MEK1/2 or GSK3β, drives the sensitivity of mESC to Tal. Cell cycle analysis of mESC following Tal treatment in 2i medium revealed a PARP1-dependent accumulation of S-phase cells (from 50% to 65%), accompanied by a corresponding reduction in the G1 population. This is consistent with reports of a compromised G1 checkpoint [42] and delayed S-phase progression due to increased replication stress in mESC [43]. A further reduced G1 population and altered S-phase progression was also observed when mESC were cultured in serum + LIF conditions but not in MEFs (Supplementary Fig. S1F), despite no sensitivity towards Tal.

From these data, we conclude that (i) HR proficient, naïve mESC are exceptionally sensitive to low-dose, single-agent treatment with PARPi, (ii) PARPi sensitivity of mESC is PARP1 dependent, (iii) PARPi sensitivity is associated with the naïve pluripotency state of mESC, and (iv) this cytoxicity is independent of cell duplication rates.

### PARP inhibition activates an ATM-dependent p53 response in naïve mESC

To further investigate the sensitivity of HR-competent naïve mESC to PARPi, we performed mRNA sequencing on mESC treated with Tal for 6, 12, or 24 h. Wild-type (wt) mESC showed pronounced transcriptional changes within the first 6 h of Tal treatment (2619 of 25 499 genes), a majority of which lasted up to 24 h of treatment (Fig. 1C and D). Most of these transcriptional changes were PARP1 dependent; *Parp1*^−/−^ mESC showed a comparably minor response to Tal treatment with most of the genes activated in wt mESC being unchanged or even downregulated in the absence of PARP1 (Fig 1C, clusters 1–3 and 5).

To identify the underlying regulatory networks, we searched for transcription factors associated with genes that were activated by Tal within the first 6 h of treatment. This yielded the tumour suppressor p53 as the most significantly enriched factor (Supplementary Fig. S1G). To ascertain the engagement of p53, we examined the Tal response of 444 genes that were previously identified as authentic p53 targets responding to doxorubicin-induced DNA-stress [44]. Of these, 219 genes (49%) were found to be upregulated within 6 h of Tal treatment (Fig. 1D), and gene set enrichment analysis confirmed a significant enrichment of p53 targets among Tal-induced genes (Normalized enrichment score = 1.29, FDR < 0.05; Supplementary Fig. S1H).

To validate p53 engagement, we assessed the phosphorylation status of p53 and the transcriptional response of the well-known p53 target genes *Fas, Tap1*, and *Wnt9a*, all of which were amongst the Tal upregulated genes detected by mRNA-seq. Immunoblot analysis revealed p53 phosphorylation at Ser15 and Ser392 after Tal treatment, comparable to TOP2A inhibition by merbarone (Fig. 1E). Analyses by reverse transcription quantitative PCR (RT-qPCR) confirmed that Tal treatment induced the p53 targets *Fas, Tap1*, and *Wnt9a* as well as the secondary target *Hey2* in a p53-dependent manner (Fig. 1F). However, p53 phosphorylation was not observed in Tal (or Ola)-treated U2OS or RPE-1 cells (Supplementary Fig. S1I), indicating that the activation of p53 by PARPi is a specific feature of mESC. The transcriptional upregulation of *Fas* and *Tap1* in response to Tal was accompanied by a PARP1-dependent enrichment of phospho-p53 at their promoters and increased deposition of H3K4me3 marks, which are consistent with gene activation (Supplementary Fig. S1J and K). These results establish that Tal treatment of mESC activates p53-dependent gene transcription.

**Figure 2.**
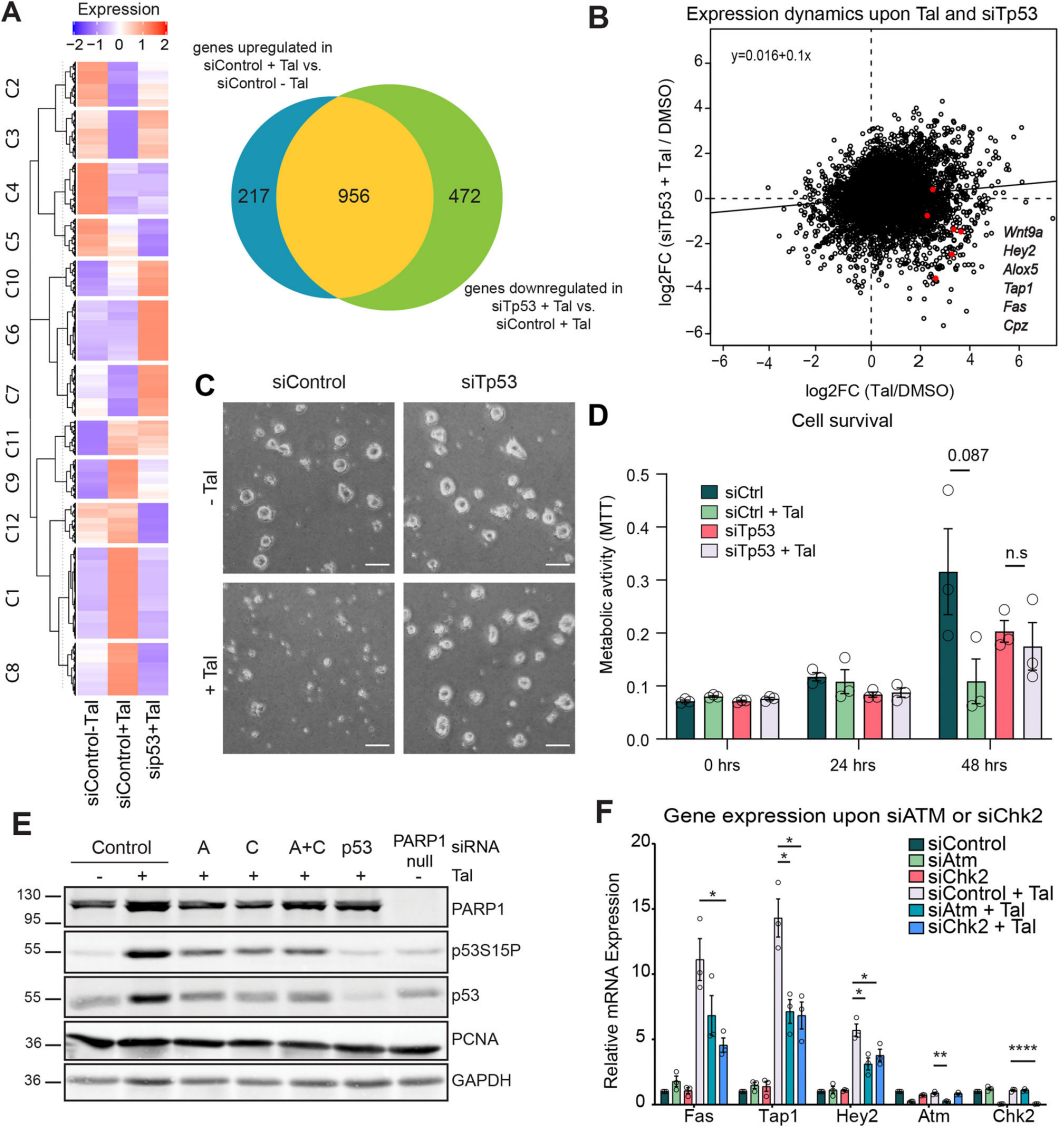
ATM-dependent phosphorylation of p53 is a central mediator of the Tal response in naïve mESC. (**A**) Left: heatmap with hierarchical clustering showing differential gene expression of siControl and siTp53-treated mESC after 5 nM Tal for 6 h. Clusters 1,8,9,11, and 12 indicate significantly upregulated genes. Right: Venn diagram overlapping dysregulated genes upon Tal in dependence of p53. (**B**) Double contrast comparing Tal-induced gene expression in control mESC (Tal/DMSO) with Tal-induced gene expression in p53-depleted mESC (siTp53 + Tal versus DMSO). Candidate genes used for validation are marked in red and names match from top to bottom. (**C**) Representative depictions of mESC sensitivity to Tal upon p53 depletion. mESC transfected with Tp53 or control siRNA were treated with 5 nM Tal for 2 days. Scale bar, 50 µm. (**D**) MTT assay showing the cell viability of mESC treated with 5 nM Tal upon siTp53 or siControl. Shown are means + SEM (*n* = 3). (**E**) Immunoblot of mESC treated with 5 nM Tal for 6 h upon ATM and/or CHK2 as well as p53 depletion by siRNA (*n* = 3). A: ATM:CHK2. (**F**) Relative mRNA expression by RT-qPCR in mESC treated with 5 nM Tal for 6 h upon ATM or CHK2 depletion (siRNA). Shown are means of relative expression + SEM (*n* = 3) versus DMSO treatment. Numbers in graphs indicate *P*-values of unpaired, two-tailed *t*-tests between conditions.

To investigate the full spectrum of p53-dependent responses to PARP1/2 inhibition, we performed total RNA-seq on mESC with or without siRNA-mediated p53 depletion (p53-directed or control siRNA) prior to exposure to 5 nM Tal for 6 h. Of the 1173 genes that were significantly upregulated by Tal in control mESC, 956 (81.5%) exhibited different regulation (downregulation or no response) when p53 was depleted (Fig. 2A). In addition to reduced Tal-induced upregulation of canonical p53 targets, p53 depletion reduced the dynamics of the global transcriptional response to Tal treatment (Fig. 2B), suggesting that p53 is a key regulator of Tal-induced stress response. In addition to cell counting and to avoid bias through cell loss during harvesting and washing, we employed a metabolic assay. This revealed that p53 depletion conferred resistance to Tal-mediated cytotoxicity (Fig. 2C and D), indicating that p53-induced gene expression mediates the observed cell death (Fig. 1A and B). Cell cycle analysis showed that siTp53 treatment reduced the accumulation of S-phase cells upon Tal from 65% in control mESC (Supplementary Fig. S1F) to 54.7%, with a concomitant decrease of the G1 population, indicating a contribution of p53 to intra S-phase checkpoint activation (Supplementary Fig. S2A)

To determine the signalling events leading to p53 activation in Tal-treated naïve mESC, we investigated the potential involvement of the upstream DNA damage response kinases ATM and CHK2 as well as ATR and CHK1. While Tal treatment did not induce typical ATR-dependent reactions, i.e. phosphorylation of CHK1 and RPA2 (Supplementary Fig. S2B), it did activate an ATM-dependent response involving the phosphorylation of H2A.X, examined as a readout for a DSB-associated DNA damage response (Supplementary Fig. S2C and D). Treatment of wt mESC with an ATM-specific inhibitor (KU-55933) [45] repressed Tal-induced phosphorylation of H2A.X, whereas ATR inhibition (AZ20) did not (Supplementary Fig. S2C and D). Depleting ATM and/or CHK2 using siRNA [46] reduced Tal-induced p53 phosphorylation (Fig. 2E) as well as p53-dependent gene activation (Fig. 2F and Supplementary Fig. S2E and F), implicating both ATM and CHK2 in p53 activation upon PARP1 inhibition.

### TET-TDG-dependent processes mediate Tal-induced cytotoxicity downstream of p53 activation

**Figure 3.**
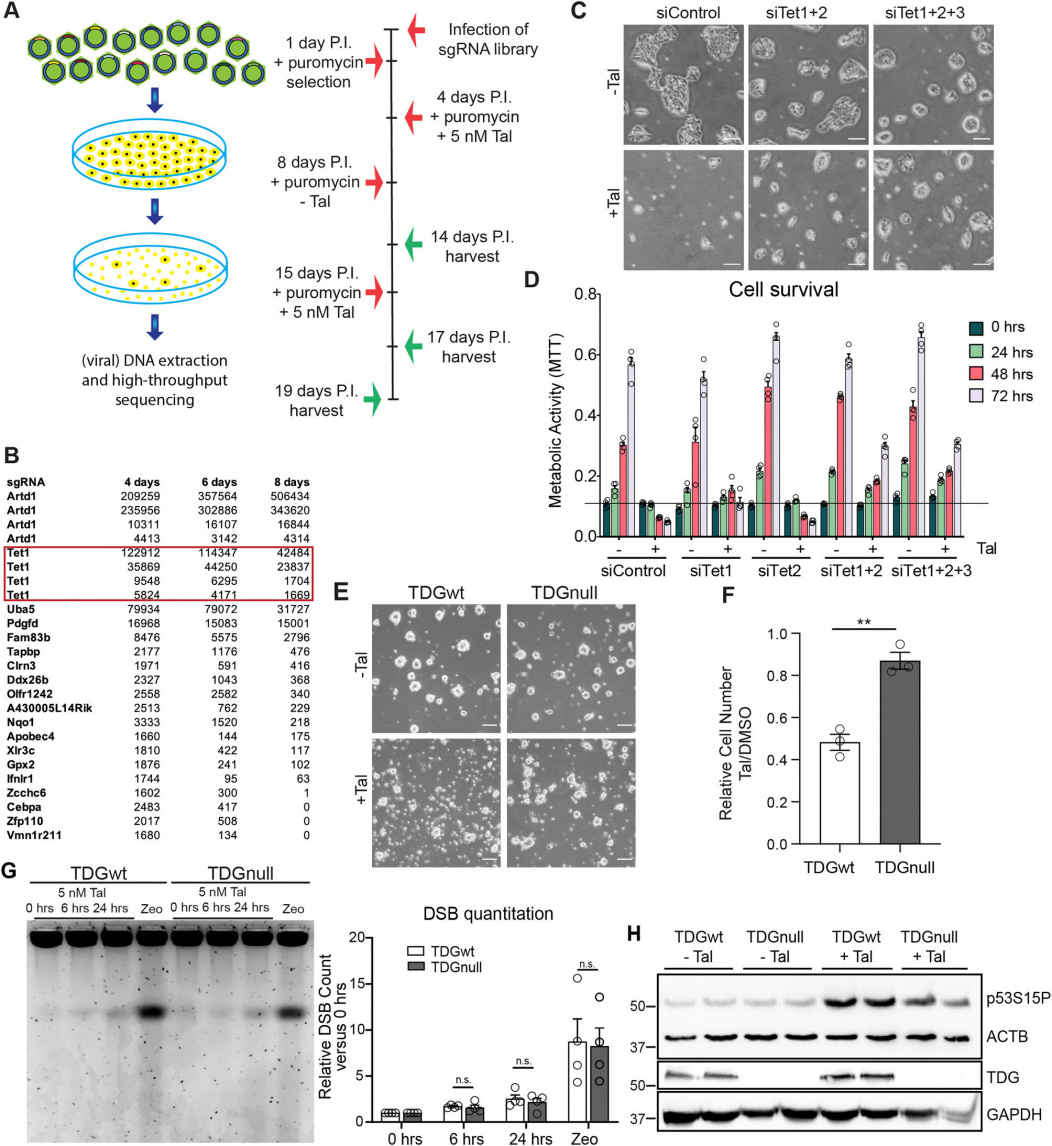
TET-TDG-mediated active DNA demethylation facilitates p53-dependent gene expression. (**A**) Schematic workflow and timeline of the CRISPR screening. Arrows indicate moments or treatment and harvesting. (**B**) Top 25 hits of the CRISPR/Cas9 screen. Individual sgRNAs were grouped according to their target gene and then ranked by the number of reads detected per sgRNA. (**C**) Representative depictions of mESC sensitivity to Tal upon TET depletion (siTet1/2/3). wt mESC transfected with indicated siRNAs were treated with 5 nM Tal for 2 days. Scale bar: 50 µm. (**D**) MTT assay showing the cell viability of wt mESC treated as in panel (C). Shown are means + SEM (*n* = 4). (**E**) Representative depictions of the sensitivity of inducible *Tdg^fl/fl^* mESC to Tal. mESC either uninduced (TDGwt) or induced with 2 µM 4OHT for 2 h (TDGnull) were treated with 5 nM Tal for 24 h. Scale bar: 40 µm. (**F**) Quantitation of viable mESC treated as in panel (E) and shown as mean + SD (*n* = 3). **P ≤ .01 by unpaired *t*-test. (**G**) Left: PFGE of inducible *Tdg^fl/fl^* mESC (TDGnull) treated with 5 nM Tal for the indicated time. Treatment with 10 µg/ml zeocin (Zeo) for 24 h served as a positive control for DSB induction. Right: quantitation of DSBs. Shown are means + SEM (*n* = 4). (**H**) Immunoblot analysis of p53 activation in *Tdg^fl/fl^* mESC either uninduced (TDGwt) or induced (TDGnull) with 2 µM 4OHT for 2 h prior to treatment with 5 nM Tal for 24 h.

To further elucidate the mechanism underlying PARPi-induced cell death, we performed a genome-wide CRISPR knockout screen (Fig. 3A) [35]. Four hundred and forty one sgRNAs prolonged cell survival upon Tal treatment in at least one of three replicate cultures. Among the 25 top hits (Fig. 3B), 2 genes were identified by all 4 targeting sgRNAs present in the library, *Parp1* and *Tet1*. While *Parp1* was an expected hit, validating the functionality of the screen, the finding of *Tet1* implied that 5mC oxidation is involved in Tal-induced toxicity. Consistently, depletion of Tet1 by siRNA (Fig. 3C and D, and Supplementary Fig. S3A and B) partially rescued mESC from Tal sensitivity. Tet2 depletion on its own had no effect but simultaneous depletion of Tet1 and Tet2 further enhanced cell survival in the presence of Tal (Fig. 3C and D). Tet3 depletion did not show an additional effect (Fig. 3D), consistent with its low expression in mESC (Supplementary Fig. S3C). To address whether TET proteins would mediate Tal sensitivity in the context of TDG/BER-dependent active DNA demethylation [47, 48], we assessed the Tal response of TDG-deficient naïve (2i) mESC, making use of an mESC line carrying a tamoxifen-inducible TDG knockout allele [49]. As previously observed with serum-cultivated mESC [50, 51], induced TDGnull mESC were hyper-resistant to Tal (Fig. 3E and F).

This effect was reproducible with an unrelated *Tdg^−/−^* mESC line complemented with a *Tdg* mini-gene (TDGwt), a catalytic dead mutant (TDGcat, N151A), or a vector control (TDGnull) [52, 53]. TDGnull as well as TDGcat mESC were hyper-resistant to Tal (Supplementary Fig. S3D). The accumulation of S-phase cells upon Tal treatment, as observed in wt mESC (Supplementary Fig. S1F), was drastically reduced (65.3% in wt and 53.9% in TDGnull), indicating a TDG-dependent intra S-phase checkpoint activation (Supplementary Fig. S3E). Curiously, this small but significant increase from 50% S-phase cells in TDGnull is similar to the one observed in siTp53 mESC (Supplementary Fig. S2A). Together, these results suggested that active TET and TDG proteins mediate Tal-induced cell death, most likely in the context of BER-mediated active DNA demethylation.

PARP1 inhibition is known to interfere with DNA repair and replication in various ways [54, 55], ultimately leading to an accumulation of DSBs during replication that then activate the observed ATM-dependent DNA damage response (Fig. 2E). Pulse field gel electrophoresis of DNA from mESC treated with Tal revealed indeed a PARP1-dependent accumulation of DSBs (Supplementary Fig. S3F). Notably, at the Tal concentrations used in these experiments, we did not detect PARP1 trapping, i.e. an enrichment of PARP1 in the chromatin fraction (Supplementary Fig. S3G [12]). We reasoned that the observed accumulation of DSB following PARPi might be induced by unrepaired lesions occurring during TET-TDG-initiated BER, which is highly active in naïve mESC [56]. However, this was not the case as TDG-depleted mESC exhibited comparable levels of Tal-induced DSB accumulation as TDG proficient cells (Fig. 3G). Similarly, p53 phosphorylation in Tal-exposed mESC was largely independent of TDG (Fig. 3H and Supplementary Fig. S3H) or TET1/2 (Supplementary Fig. S3I). Likewise, p53 activation by zeocin (Supplementary Fig. S3H), which we used as a control to induce DNA damage that does not initiate TDG-dependent repair [57], was functional and thereby independent of TDG. Together, these results demonstrate that TET and TDG depletion rescues Tal-induced cytotoxicity without affecting DSB accumulation or p53 activation. We therefore conclude that TET-TDG-dependent processes contribute to intra S-phase checkpoint activation and cell death downstream of DNA damage signalling and p53 activation.

### TET-TDG-mediated active DNA demethylation facilitates p53-dependent gene expression

**Figure 4.**
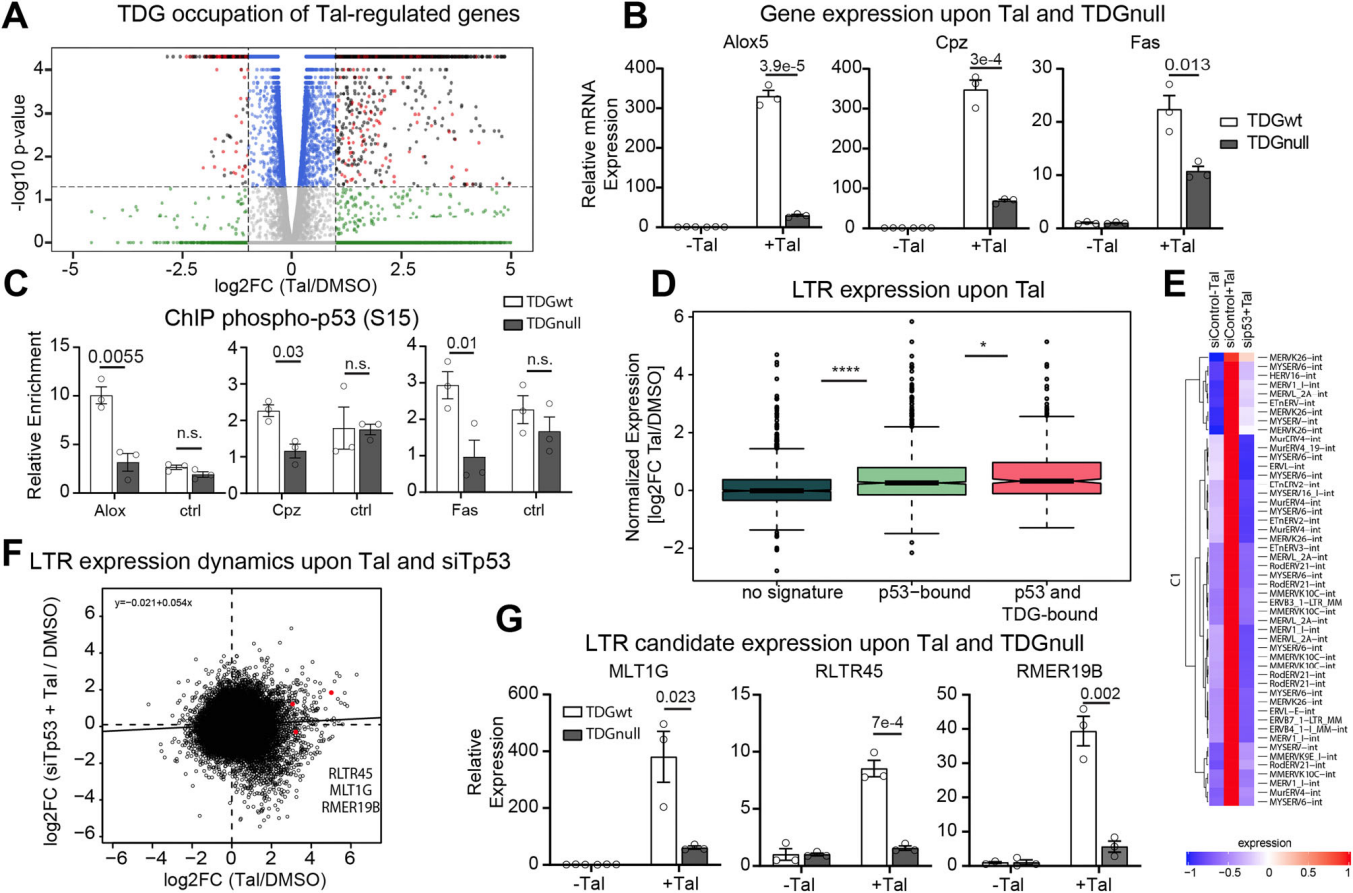
Tal induces ERV expression in a p53- and TDG-dependent manner. (**A**) Volcano plot depicting differentially expressed genes upon 6 h of Tal treatment of wt mESC. Red dots indicate significantly Tal-regulated genes (*n* = 1859) and anthracite dots indicate significantly Tal-regulated genes occupied by TDG at the promoter and/or in the gene body [58] (*n* = 1594). (**B**) RT-qPCR analysis in wt or Cre-induced *Tdg*^fl/fl^ mESC treated with 5 nM Tal for 24 h. Shown are means + SEM (*n* = 3). (**C**) ChIP-qPCR analysis of phospho-p53 in *Tdg*^fl/fl^ mESC treated with 5 nM Tal for 24 h. Shown are mean fold enrichments (% input) in Tal versus DMSO treated cells (+Tal/−DMSO) + SEM (*n* = 3). A non-targeted region, ∼120 kb upstream of *Chordc1*, is shown as a negative control. (**D**) Expression of detected LTRs without p53 or TDG binding (random subset *n* = 1397), LTRs with a p53 binding site within the sequence (p53-bound, *n* = 1397), and LTRs, co-occupied by p53 and TDG (*n* = 507). (**E**) Heat map showing expression levels of the top 50 ERVs deregulated after 6 h of Tal treatment in siControl and siTp53-treated mESC. (**F**) Double contrast of gene expression comparing p53-dependent, Tal-induced LTR expression (siTP53 + Tal versus DMSO) with Tal-induced LTR expression (Tal versus DMSO). Validated candidate repeats are indicated in red and labelled from top to bottom. (**G**) RT-qPCR analysis in wt or Cre-induced *Tdg*^fl/fl^  *Tdg*^fl/fl^ mESC treated with 5 nM Tal for 24 h. Shown are means + SEM (*n* = 3). Asterisks, *P ≤ .05, **P ≤ .01, ***P ≤ .001, ****P ≤ .0001 by unpaired, two-tailed *t*-tests.

Since TETs and TDG mediate Tal-induced cell death downstream of p53 activation, we investigated whether active DNA demethylation controls p53-dependent gene expression upon Tal treatment. Of the 1859 genes upregulated more than two-fold upon 6 h of Tal treatment, 1594 showed TDG enrichment in their promoters and/or gene bodies (Fig. 4A) [58], suggesting a contribution of TDG to the regulation of Tal-induced genes (86%, χ^2^  *P* = 1.87e^−17^).

To specifically investigate TDG’s role in p53-dependent gene activation upon Tal treatment, we examined the three p53 target genes *Alox5, Cpz*, and *Fas*. Consistent with the mRNA-seq and total RNA-seq data, RT-qPCR showed a strong Tal-dependent activation of these genes in wt mESC. This induction was significantly lower when TDG was absent, inactive (Supplementary Fig. S4A), or conditionally deleted by CRE (Fig. 4B), suggesting that TDG activity facilitates the activation of specific p53 target genes in response to Tal. This is in line with previous observations, indicating that TDG facilitates p53-dependent transcription more generally [59]. Notably, the depletion of TET1 and TET2 in addition to TDG did not further reduce the Tal inducibility of these genes when compared to their individual depletions (Supplementary Fig. S4B), consistent with an epistatic functional interaction between TETs and TDG. Consistently, chromatin immunoprecipitation (ChIP) analyses showed for all p53 target genes examined (*Alox5, Cpz*, and *Fas*) a positive enrichment of TDG (Supplementary Fig. S4C) and p53 at their promoters (Fig. 4C) and, importantly, Tal-induced p53-association with these promoters was dependent on TDG (Fig. 4C). Similar to recent observations on cell cycle-driven gene control [60], these findings demonstrate that, following p53 activation by Tal, TDG activity is required to facilitate its binding to target gene promoters.

### PARP inhibition induces ERV expression in a p53- and TDG-dependent manner

It has been reported that DNA damage derepresses transposable genomic elements by engaging p53 [61]. To assess whether transposon activation by p53-dependent DNA stress responses may contribute to Tal-induced cell death in mESC, we re-examined the total RNA-seq data for the expression of repetitive genomic elements. We found that RNAs derived from retrotransposable elements, including LTRs as well as long and short interspersed nuclear elements (LINEs, SINEs), were indeed significantly increased upon Tal treatment (Supplementary Fig. S4D). Most prominent was the upregulation of ERVs, a subgroup of LTR-containing repeats. Consistent with the presence of p53 binding motifs in ERVs [62, 63], LTRs exhibited a higher p53 occupancy following DNA stress induction (with doxorubicin [44]) than other TEs in mESC (Supplementary Fig. S4E). LTRs exhibiting TDG occupation and stress-induced p53 binding were more strongly upregulated by Tal treatment than LTRs not associated with either p53 or TDG (Fig. 4D). The upregulation of the top 50 differentially expressed ERVs (Fig. 4E), and in fact, most of the differentially expressed LTRs (totally 2337), was clearly p53-dependent (Fig. 4F and Supplementary Fig. S4F and G). RT-qPCR analysis of three candidate LTRs showing deregulation in RNA-seq (MLT1G, R LTR45, RMER19B) confirmed the active role of p53 in Tal-induced transcriptional derepression (Supplementary Fig. S4H). Likewise, TDG disruption strongly reduced Tal-induced ERV expression (Fig. 4G and Supplementary Fig. S4I). Combined depletion of TDG, TET1, and TET2 did not further decrease Tal-mediated ERV induction (Supplementary Fig. S4J), consistent with an epistatic function in p53-dependent ERV expression. Together, these data provide strong evidence that PARP1 inhibition in naïve mESC induces the expression of TEs, particularly LTR-containing ERVs, in a p53- and TET-TDG-dependent manner.

### p53-dependent ERV activation coincides with 5mC oxidation and DNA SSB formation by TET-TDG -BER

To address the mechanism underlying TET-TDG-facilitated transcription at TEs, we first measured DNA methylation levels at two ERVs and the canonical p53 target *Fas*. Bisulphite-pyrosequencing across selected CpG sites showed an average of 40%–60% methylation at RLTR45 and RMER19B and 5%–10% methylation at the *Fas* gene (Supplementary Fig. S5A). Treatment with Tal and/or depletion of TDG did not detectably alter the 5mC states at these sites. 5mC derivatives, analysed globally by mass spectrometry, showed the expected increase of 5fC and 5caC following TDG disruption but no change in 5hmC (Supplementary Fig. S5B–D). Tal treatment increased the global levels of 5hmC, as reported before [64], as well as those of 5fC and 5caC, but only in TDG-deficient cells. These results show that Tal induces TET-TDG-dependent DNA demethylation activity in mESC but does not detectably alter the steady-state levels of 5mC at ERVs induced by Tal.

**Figure 5.**
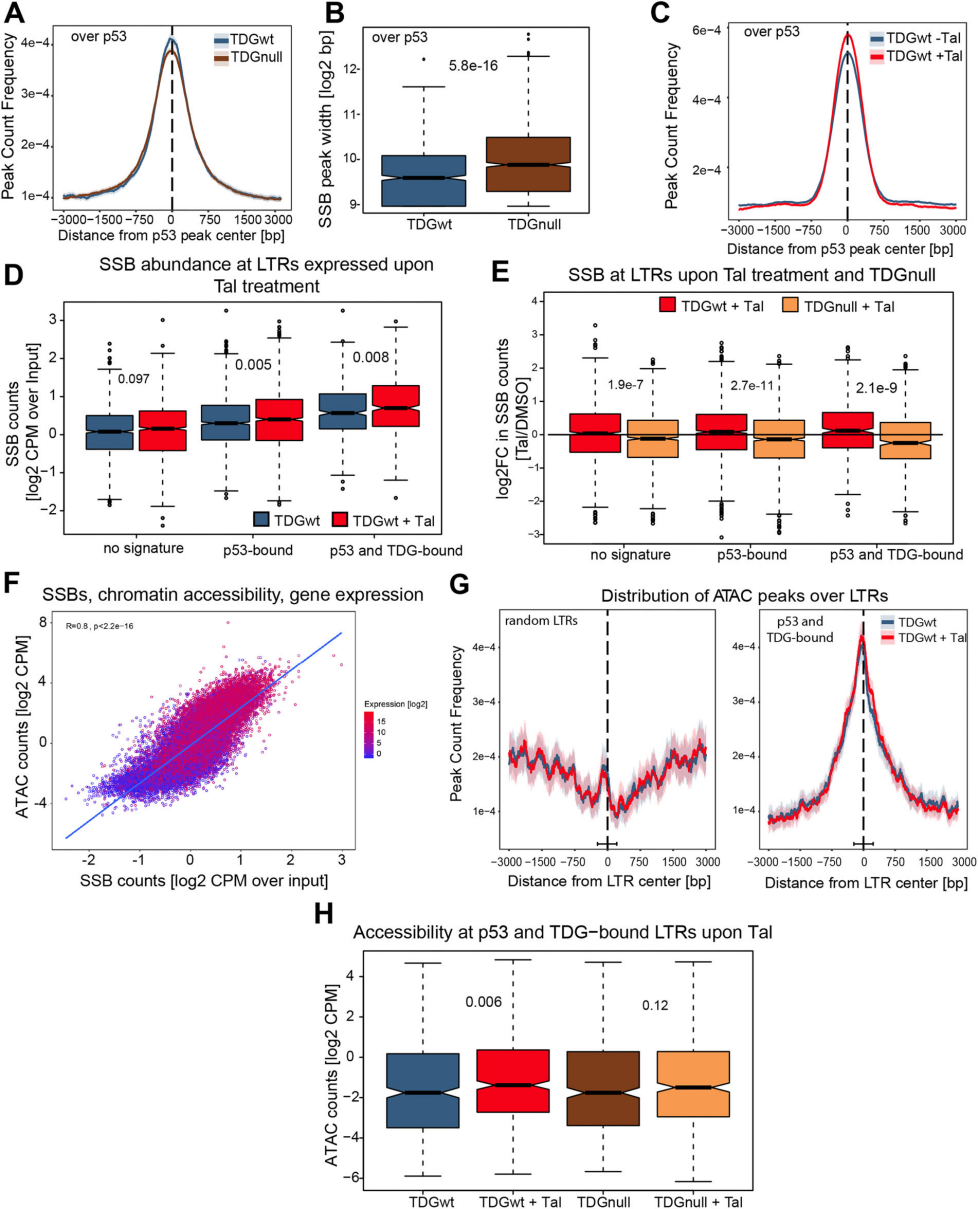
Tal treatment induces SSB at ERVs in a TDG-dependent manner. (**A**) Comparison of SSB-seq peak density in *Tdg*^fl/fl^ mESC with or without 4OHT-induced TDG depletion. Distribution of all detected SSB-seq peaks centred around identified TDG (left) [58] or p53 sites (right) [44]. Shadings indicate the 95% confidence interval. (**B**) Extension in of SSB peaks in bp, coinciding with TDG (left) or p53-bound regions (right). (**C**) Comparison of SSB-seq peak density in wt mESC with or without Tal treatment, 3 kb around the centre of TDG (left) or p53 peaks (right) as in panel (A), Shadings indicate 95% confidence intervals. (**D**) SSB-seq signal as TMM-normalized log2 CPM at a random subset of detected LTRs without p53 or TDG binding (no signature, *n* = 1397), with a p53 binding site (p53-bound, *n* = 1 397), or co-occupied by p53 and TDG (*n* = 507). (**E**) SSB-seq signal at expressed LTRs from panel (D) in Tal-treated *Tdg*^fl/fl^ ESCs with or without prior TDG deletion. Sown are log2FC Tal versus DMSO. (**F**) Correlation of SSB-seq (*x*-axis) and ATAC-seq (*y*-axis) signals as TMM-normalized log2 CPM at promoters of all detected genes (TSS +500 bp/−2 kb, *n* = 23 087) in wt mESC. Pearson correlation coefficient is indicated for the linear regression. Genes are colour-coded by their absolute expression level. (**G**) ATAC-seq peak density at randomly selected LTRs (left, *n* = 1397) or p53 and TDG-bound LTRs (*n* = 507) in *Tdg*^fl/fl^ mESC with or without Tal treatment. Lighter shadings indicate the 95% confidence interval. (**H**) TMM-normalized log2 ATAC-seq CPM at p53 and TDG-bound LTRs. Asterisks: *P ≤ .05, **P ≤ .01, ***P ≤ .001, ****P ≤ .0001 by unpaired, two-tailed *t*-tests.

In the absence of changes in DNA methylation, we reasoned that TET-TDG-dependent SSB formation in the context of dynamic DNA methylation (cycling DNA methylation-demethylation) may facilitate the transcriptional activation of ERVs. Analysing published SSB-seq data from wt and TDG-deficient mESC, both treated or not with Tal [12], revealed an over-representation of CpGs in SSB-enriched DNA fragments when compared to the input. This indicates a preferred association of SSBs with DNA cytosine methylation (Supplementary Fig. S5E). SSB-containing fragments were enriched in genic and gene regulatory regions, including enhancers, promoters, and 5′UTRs (Supplementary Fig. S5F), as previously observed [36, 65, 66]. Notably, SSB peaks showed a strong co-occurrence with published p53 enrichment sites in doxorubicin-treated mESC [44] (Fig. 5A).

In TDG-depleted mESC, SSB enrichment at p53-occupied sites was significantly reduced, yet still present, and characterized by significantly extended peak areas (Fig. 5B), indicating the involvement of alternative mechanisms with different targeting properties. These findings suggest that TDG and p53 cooperate in gene regulation through targeted SSB formation. Upon Tal treatment of mESC, we observed a significant increase in SSBs at p53-binding sites induced by DNA stress, consistent with TDG-generated SSBs playing an active role in the p53-dependent response to PARPi (Fig. 5C).

Considering these global findings, we proceeded to analyse the occurrence of SSBs at expressed LTR regions. Indeed, LTR-associated SSBs increased in response to Tal, particularly at p53- and p53-TDG co-occupied LTRs (Fig. 5D and Supplementary Fig. S5G and H). The Tal-induced formation of SSBs at expressed LTRs was TDG-dependent; in the absence of TDG, SSB levels were significantly reduced (Fig. 5E). This indicates that TDG-BER-mediated SSBs contribute to the efficient regulation of transcription upon Tal-induced p53 activation. To investigate whether Tal-induced, p53-dependent expression of LTRs is facilitated by SSB formation and subsequent chromatin change [67], we performed ATAC-seq in wt and TDG-depleted mESC treated with Tal. Globally, SSB abundance correlated directly with chromatin accessibility at gene promoters and the expression of the corresponding genes (Fig. 5F). ATAC-seq peaks also coincided with expressed LTRs that are co-occupied by p53 and TDG but not with LTRs not bound by either protein (Fig. 5G). Quantitation (at read level) of chromatin accessibility upon Tal at LTRs co-occupied by p53 and TDG revealed a TDG-dependent increase in the ATAC signal (Fig. 5H), which was not significant at randomly selected LTRs without p53 or TDG binding (Supplementary Fig. S5I). Based on these results, we concluded that the p53- and TET-TDG-induced transcriptional Tal response at selected LTRs involves SSB formation and a coincident increase in chromatin accessibility. However, as expected, the effects on chromatin accessibility were not dramatic for naïve mESC as their genome is generally in a highly accessible state [68]. Taken together, these data provide strong evidence for a role of TDG-initiated BER in facilitating p53-dependent activation of LTR transcription in Tal-treated mESC.

### PARPi-triggered ERV expression induces a viral mimicry response in mESC

**Figure 6.**
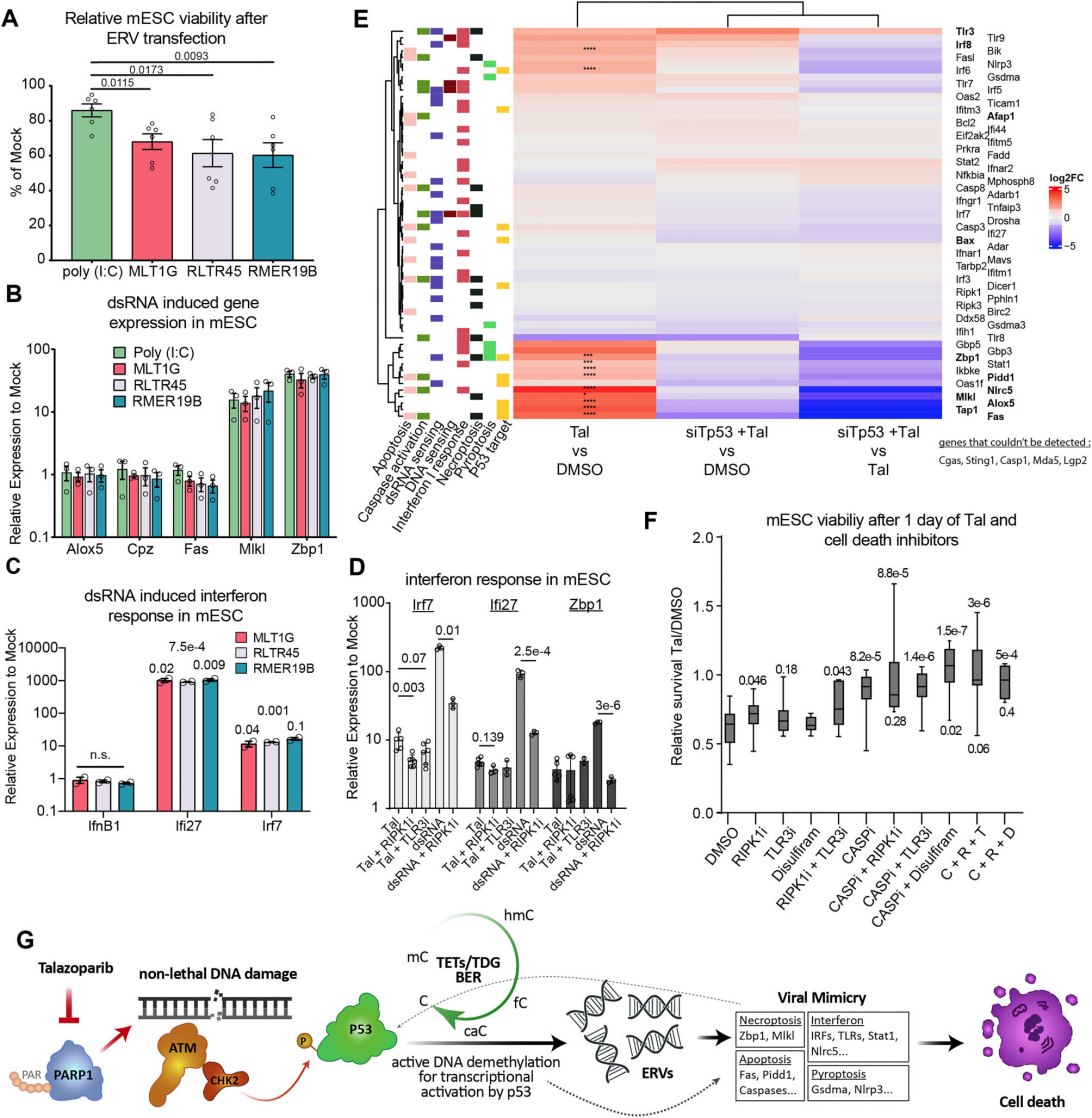
Tal-mediated ERV derepression causes viral mimicry. (**A**) Percentage of trypan blue-negative mESC treated with *in vitro* transcribed ERV dsRNA as indicated, 2 days after transfection (1 µg/ml medium). Shown are means with SEM (*n* = 6). (**B**) Relative mRNA expression (RT-qPCR) of canonical p53 targets and necroptosis markers in mESC treated as in panel (A). Shown are means + SEM (*n* = 3). (**C**) Relative mRNA expression (RT-qPCR) of known interferon-responsive genes in mESC cells after treatment as in panel (A). Expression of *Ifi44* was not detectable. All error bars represent the SD of *n* = 2 experiments. The numbers above bars indicate the *P*-value of unpaired *t*-tests compared to transfection control. (**D**) Relative mRNA expression (RT-qPCR) of interferon signalers and Zbp1 upon indicated treatments. Shown are means ± SEM (*n* = 3–6). (**E**) heatmap (based on total RNA-seq) of genes of interest upon Tal and/or siTp53. Left: non-exhaustive categorization of genes according to pathways. Genes in bold are mentioned in the text and are of special interest. *P < .05, ***P < .001, ****P < .0001 in the comparison Tal versus DMSO. (**F**) WST-assay displaying metabolic activity of mESC treated with Tal (5 nM) for 1 day after 1 day of pre-incubation and following co-incubation for 1 day with RIPK1i (necrostatin-1, 60 µM), TLR3i (CU-CPT-4a, 7.5 µM), CASPi (Z-VAD-FMK, 25 µM), and disulfiram (200 nM). The number above the boxes indicates the *P*-value of unpaired *t*-test comparing the condition to DMSO. The number below indicates the results of an unpaired *t*-test comparing the condition with CASPi alone. No number indicates *P*-value >.5. *n*(C + N + D) = 4, *n*(others): 8–12. (**G**) Summarizing model: Tal inhibits PARP1, which creates TDG-independent DNA DSBs. ATM activates p53 by phosphorylation, which in turn causes the transcription of canonical p53 targets, as well as the derepression of TEs; both processes are dependent on TET-TDG-mediated active DNA demethylation. dsRNA is detected by the mESC and elicits an innate immune response that comprises multiple p53-dependent cell death pathways.

We next addressed whether increased ERV expression can account for the observed cytotoxicity of Tal in mESC. Gene set enrichment analyses of the Tal-induced differentially expressed genes (based on total RNA-seq data) revealed a significant representation of the type 1 interferon response pathway and a trend towards necroptosis-related genes (Supplementary Fig. S6A and B). Upon depletion of p53, both pathways were significantly underrepresented in the transcriptional response to Tal (Supplementary Fig. S6C). Notably, both of these pathways are known to respond to dsRNA infection [69, 70]. To address whether ERVs can induce interferon-like responses and/or necroptosis, we transfected mESC with *in vitro*-transcribed dsRNAs (Supplementary Fig. S6D). Transfection of ERV dsRNA significantly reduced the viability of naïve mESC when compared to mock transfections or control transfections with equal amounts of dsRNA-mimicking poly-inosinic:cytidylic acid (poly-I:C) (Fig. 6A and Supplementary Fig. S6E). This cytotoxic response was accompanied by an upregulation of *Mlkl* and *Zbp1*, both components of the necroptosis pathway and induced upon Tal treatment, whereas no transcriptional upregulation of canonical p53 targets was detected (Fig. 6B). Two out of four interferon-response genes examined (*Irf7* and *Ifi27)* were explicitly activated in mESC transfected with ERV dsRNAs (Fig. 6C), while one (*Ifi44*) was not detectable. To examine the dependency of these transcriptional responses on necroptotic signalling via RIPK1/RIPK3 and/or sensing of dsRNA via TLR3, we examined these responses in the presence of a RIPK1-inhibitor (Necrostatin-1) and a TLR3 inhibitor (CU-CPT4a), respectively. RIPK1 inhibition significantly reduced *Irf7* induction upon Tal and dsRNA transfection (Fig. 6D) [71], linking the activation of necroptosis with the interferon response. TLR3 inhibition showed a trend towards reduced *Irf7* induction upon Tal but did not reach statistical significance. Together, these results demonstrated the competence of mESC to activate necroptosis and a partial, cell-intrinsic interferon response by sensing Tal-induced dsRNAs, presumably via ZBP1. The results also corroborate the reported inability of mESC to establish a full-fledged interferon response upon viral infection [24, 26], as compared to HT-29 cells (Supplementary Fig. S6F and G) [72].

Combined activation of type I interferon and necroptosis signalling as well as the upregulation of the dsRNA sensors *Tlr3* and *Zbp1* were implicated as the mechanism responsible for Tal-induced toxicity in mESC (Fig. 6E). Inhibiting necroptosis via RIPK1 inactivation indeed yielded improvement in mESC viability after 1 day of Tal treatment (∼+16%) but inhibiting TLR3 had no such effect (Fig. 6F). Combined inhibition of both did not further increase the viability of mESC.

As genes related to apoptosis, including *Fas, Afap1, Pidd1*, and *Nlrc5*, which have been associated with caspase activation [73, 74], were also upregulated upon Tal treatment (Fig. 6D and Supplementary Fig. S6H), we tested the effect of caspase inhibition on Tal toxicity. Pan-caspase inhibition (Z-VAD-FMK, CASPi) significantly increased mESC survival after 1 day of treatment (∼+40%; Fig. 6F), suggesting that caspase activity contributes to Tal’s toxicity to mESC. Consistently, we observed PARP1 cleavage upon Tal treatment (Supplementary Fig. S6I), which, however, was stimulated rather than inhibited by CASPi. Notably, although mESC were more viable and metabolically active when treated with CASPi in addition to Tal (Fig. 6F), they appeared as unhealthy by morphological criteria as mESC treated with Tal alone (Supplementary Fig. S6J). Along with increased levels of p53 phosphorylation (Supplementary Fig. S6I) this indicates an ongoing cellular crisis, which is also reflected by the cell death observed 2 days into Tal treatment despite CASPi (Supplementary Fig. S6K). Combined treatment of mESC with CASPi and either TRL3i or RIPK1i had no significant additional effect on cell survival (Fig 6F). Moreover, since the genes of the pyroptosis pathway, *Nlrp3* and Gasdermin A (*Gsdma*), were also upregulated in Tal-treated mESC (Fig. 6E), we investigated the contribution of pyroptosis to Tal-induced toxicity in mESC by inhibiting pore formation with disulfiram [75]. Disulfiram alone did not show an effect on mESC survival upon Tal treatment but increased survival additively in combination with CASPi (Fig. 6F and Supplementary Fig. S6K).

Together, these data provide multiple lines of evidence that Tal-mediated activation of ERV transcription induces a cell death program in mESC. This involves the activation of a non-canonical interferon response by dsRNA sensing and signalling through TLR3 and ZBP1 [76, 77], and of programmed cell death pathways, including apoptosis and pyroptosis. These p53-, TET-, and TDG-dependent cellular responses to dsRNA stress strongly resemble viral mimicry [18, 19], a cell elimination pathway that may be of importance in mESC that lack a conventional interferon response [23, 26].

## Discussion

PARP inhibitors (PARPi) are being used in the treatment of HR-deficient cancers [78], yet the effects of PARPi on non-cancerous cells are still only partially understood. We show here that naïve pluripotent mESC, proficient in HR-mediated double-strand break repair, and therefore expected to be PARPi resistant, are hypersensitive to PARP1 inhibition; FDA-approved PARPi Tal and olaparib induced PARP1- and p53-dependent cell death in mESC (Fig. 1A–D). Mechanistically, inhibition of PARP1 in mESC induces a DNA damage response involving p53-mediated and TET-TDG-dependent aberrant expression of ERVs, which then elicits a complex cell death program involving necroptotic, interferon-related, and caspase-activating responses (Fig. 6G).

The observed toxicity of PARPi in mESC is associated with naïve pluripotency, a cell state that is attributed to the unique structure and dynamic properties of chromatin. Genomes of naïve mESC are globally DNA hypomethylated [13] and highly accessible [68], and therefore more permissive for aberrant transcription than differentiated and epigenetically restricted cells [79]. Our data support the notion that this difference in overall chromatin accessibility can explain the sensitivity of mESC to PARP1 inhibition. Cell duplication rate sometimes associated with PARPi sensitivity is, however, not a determining factor as Tal-resistant mESC show the same (TDGnull) or even faster (PARP^−/−^) cell duplication time than sensitive wt mESC, and mESC cultivated in 2i-medium duplicate more slowly but are more sensitive to PARPi than when kept in serum + LIF conditions (Supplementary Figs S1 and S3).

Despite the globally hypomethylated state of genomes in naïve mESC, localized TET-TDG-mediated dynamic DNA methylation is still ongoing, contributing to the establishment of a dynamic transcriptional state required for faithful lineage commitment and differentiation [48, 51, 80, 81]. Active DNA demethylation involves the formation of SSBs through BER, thus generating substrates for PARP1 binding and activation [12, 51, 82]. Given these observations, the cytotoxicity of PARPi in mESC could therefore be explained by the accumulation of toxic amounts of unrepaired SSBs (and, consequently, DSBs) generated through TET-TDG-BER-mediated DNA demethylation. Two lines of experimental evidence, however, implicate a different and unexpected mode of action where the role of TET and TDG is downstream, rather than upstream, of the primary DNA damage response induced by PARPi: (i) TDG depletion in mESC did not affect PARPi-induced DNA DSB accumulation but rescued the cytotoxicity of PARPi (Fig. 3G), and (ii) PARPi activated p53 irrespective of the presence or absence of TET (Supplementary Fig. S3I) or TDG (Fig. 3H and Supplementary Fig. S3H).

These findings show that Tal-induced cytotoxicity in HR proficient mESC is not primarily a consequence of TET-TDG-dependent DNA strand-break formation, but of TET-TDG-dependent transcriptional processes initiated downstream of an ATM and p53-dependent DNA damage response.

Loss of p53 function has been demonstrated to sensitize cancer cells to PARPi by disrupting checkpoint enforcement, thereby enabling the unrestrained accumulation of lethal DNA damage following deoxy-uridine treatment [83]. In contrast, DSB levels accumulating in TDGnull mESC upon PARPi are tolerated but activate a p53 response, which finally causes cell death. Notably, reinstating p53 functionality to restore cell-intrinsic pathways for cell death has been observed in other cell types [84]. Supporting a role for TET-TDG activity downstream of activated p53, genome-wide gene expression and DNA SSB analyses showed that Tal-induced transcription of p53 targets coincided with the formation of TDG-dependent DNA SSBs at these loci (Fig. 5).

Tal-induced gene activation, despite involving TET and TDG activities, had no obvious effect on the local steady-state levels of DNA methylation (Supplementary Fig. S5A), indicating that 5mC turnover rather than the level of the modification itself is relevant for the observed transcriptional responses. This is in line with a previous report describing high localized DNA demethylation activity and 5mC turnover closely related to expression levels despite the already globally hypomethylated and well-accessible genome of mESC [85]. We have recently published a report, describing how PARPi via Tal can inhibit TDG-BER-mediated SSB formation, based on covalent PARylation of BER proteins [12]. This might, at first sight, seem contradictory to what we describe here. It is therefore important to underline that in the previous study we described the effect of Tal on repair of genomic sites that already undergo active DNA demethylation in an unchallenged steady-state. On the contrary, here, we shed light on different sites that are responding *de novo* to damage-induced signalling and are therefore subject to high transcriptional initiation.

The treatment of mESC with Tal induced canonical p53 target genes but also strongly upregulated the expression of LTR-containing ERVs. This coincided with the activation of genes involved in the type I interferon response [63, 86] and necroptosis, which could be phenocopied by the transfection of *in vitro*-transcribed and reconstituted ERV dsRNAs (Fig. 6). We therefore propose that Tal induces aberrant expression of ERVs in mESC, generating dsRNAs that then activate an interferon-like response as well as cell death pathways that include caspase activity and necroptotic signalling. While the mechanistic resolution of pathways leading to Tal-mediated cell death in mESC still requires more experimentation, the overall and predominant response of mESC to PARPi strongly resembles conditions described as viral mimicry [87]. Viral mimicry refers to a phenomenon first observed in cancer cells where DNA stress and damage signalling (involving p53) causes the derepression of endogenous viral elements, generating dsRNAs that then activate an innate immune response and cell death through necroptosis and pyroptosis pathways with caspase activation comparable to PANoptosis [88]. Notably, viral mimicry was observed to be more pronounced upon inhibition of DNA and histone methylation, implicating an important role of accessible chromatin state [18, 19, 87], a feature that generally applies to chromatin in pluripotent stem cells, including mESC. Regarding the dsRNA sensor involved in the activation of the observed interferon-like response in Tal-treated mESC, a wide range of factors must be considered. However, our data implicate ZBP1 and possibly also TLR3 and NLRP3, but not MDA5 or LGP2 (Fig. 6). Notably, the signalling pathways triggered upon Tal-induced viral mimicry in mESC appear to be complex, as indicated by the concomitant upregulation of TLR3 and NLRC5, of which the latter has been reported to inhibit TLR3-signalling in turn [89]. A recent report revealed increased cell death upon PARPi after viral infection of cancer cells, due to reduced PARylation of death-inducing signalling complex (DISC) components [90]. While mESC are incompetent of interferon signalling [24], the modulation of DISC activity through interference with PAR levels should be taken into consideration in follow-up work. Another very recent study showed that MLKL and the necrosome undergo PARylation, which facilitates MLKL phosphorylation and hence oligomerization to execute necroptosis [91] These findings may serve as an explanation for why we observe a strong upregulation but no phosphorylation of MLKL upon Tal in our mESC model. Further investigation will be necessary to dissect the molecular pathways contributing to Tal-induced viral mimicry and mESC toxicity.

In general, our findings establish the role of p53-targeted, TET-TDG-dependent 5mC oxidation and repair in mediating transcriptional stress responses, which, in the context of permissive stem cell genomes extend to the overexpression of ERVs [23, 92, 93]. Viral mimicry, involving interferon-like and necroptotic responses, may then be a possibility for stem cells to mitigate the genome-destabilising effects of non-physiological ERV activation.

What we describe here for embryonic stem cells may occur also in subpopulations of cancer cells. Certain cancer cells, including those undergoing epithelial-to-mesenchymal transition, exhibit high phenotypic plasticity and can assume stem cell-like properties in terms of chromatin organization and gene expression [94]. Such stem cell-like cancer cells may thus respond to a PARPi-based therapy with viral mimicry and cell death, irrespective of the tumour’s HR status, depending on the activity of PARP1, p53, TET, and TDG, thereby limiting tumour heterogeneity and metastasis. Defects in either of these genes, however, would be expected to confer PARPi resistance to this ‘aggressive’ subpopulation of cancer cells. A recent study reported enhanced sensitivity of HR-competent acute myeloid leukaemia cells to PARPi when co-cultured with vitamin C [95], which is well-known to stimulate TET-TDG-dependent active DNA demethylation. Based on our findings, we can infer that the combination of PARPi with enhanced DNA demethylation may trigger viral mimicry and enhanced cell death, as observed in cancers treated with DNA- or histone demethylating agents [96], or high-dose vitamin C [97]. Finally and importantly, non-cancerous cells with high TET activity and stem cell features, such as neuronal progenitor cells [98], may also respond to PARPi through viral mimicry, which may be worth considering when assessing the causes of adverse side effects of the anti-cancer therapy.

Further investigation will have to address the molecular and mechanistic underpinnings of the complex network of genomic responses that induce viral mimicry and cell death in stem- and differentiated cells upon PARP1 inhibition. Additionally, whether viral mimicry is a PARPi-specific response of stem cells or a more general response to any form of cell stress will need to be clarified. Our work provides fundamental insights into the biology of genome–environment interactions, particularly the role of viral mimicry in stress and cell death regulation. It raises mechanistic questions that will help resolve the understanding of the long-term effects on cell- and tissue homeostasis of cell stress-inducing cancer therapies, whether targeted or conventional.

## Supplementary Material

gkag537_Supplemental_File

## Data Availability

The SSB-seq and ATAC-seq data are available on gene expression omnibus (GEO) under the accession number GSE166964, the RNA-seq in the bioproject PRJNA743896, and CRISPR-screen data are accessible in the bioproject PRJNA1279667. Other raw data can be provided upon request.
