# Supplementary material for: PARP1 inhibition in naïve mouse embryonic stem cells induces viral mimicry
Source: Nucleic Acids Res. 2026 Jun 3;54(10):gkag537. doi: 10.1093/nar/gkag537 (PMC13232494; doi:10.1093/nar/gkag537)
Supplement: gkag537_Supplemental_File [file gkag537_supplemental_file.pdf]

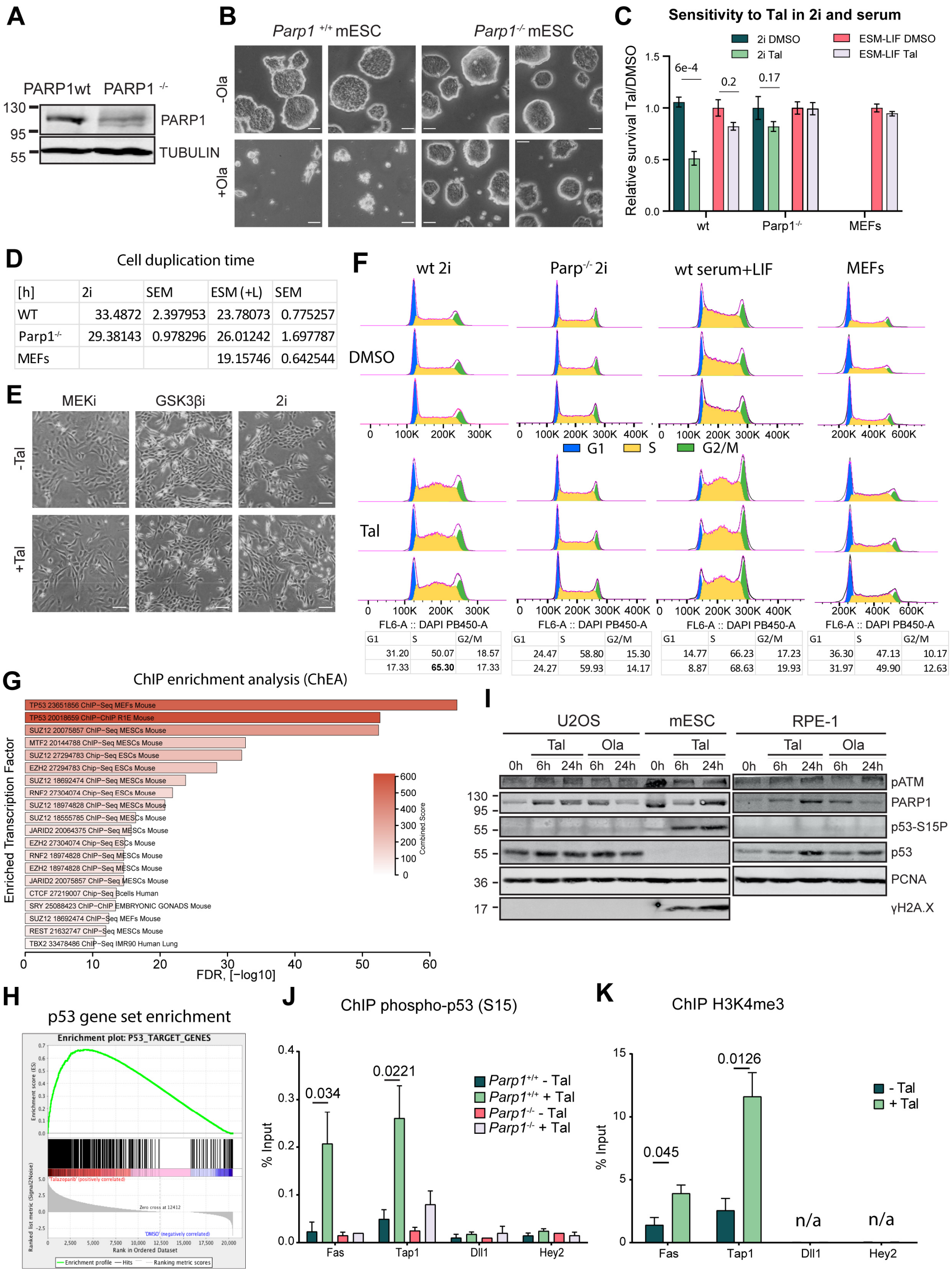

Supplementary Figure 1. Related to Figure 1; PARP1 inhibition is toxic in naïve mESC in a p53-dependent manner

(A) Immunoblot analysis of PARP1 disruption efficiency.
(B) Representative pictures show the sensitivity of mESC to Olaparib (Ola). WT and *Parp1*$^{-/-}$ mESC were treated with 10 µM Ola for 3 days. Scale bar represents 20 µm.
(C) WST8 assay showing the relative metabolic activity approximating cell viability of wt and *Parp1*$^{-/-}$

mESC grown in either 2i or embryonic stem cell medium with serum (ESM) + LIF, as well as MEFs upon 5 nM Tal treatment for 24 h. Shown are mean + SEM (n=3-4). Numbers above boxes indicate P-values of two-tailed unpaired t-tests.
(D) Cell duplication time of indicated cell lines in 2i and ESM + LIF as mean and SEM (n=3-7)
(E) Representative pictures show NIH 3T3 cells incubated with MEKi and GSK3βi either alone or together. Cells were treated with 5 nM Tal for 2 days. Scale bar represents 50 µm.
(F) Cell cycle analysis of indicated clones in 2i medium or with serum (MEFs) with or without 5 nM Tal treatment for 24 h. Relative population per cell cycle phase are indicated as %, mean (n=3). Black lines represent the actual cell counts, pink lines indicate the sum of all modelled subpopulations. Below: Quantification of subpopulations based on the Watson (pragmatic) model.
(G) Enrichment of transcription factor binding motifs (ChEA) associated with upregulated genes upon treatment with 50 nM Tal for 6 h.
(H) Gene set enrichment analysis of p53-bound genes in mRNAseq of wt mESC in response to treatment with 50 nM Tal for 6 h.
(I) Immunoblot analysis of U2OS, human RPE-1 cells and wt mESC treated with 5 nM Tal or 10 µM Ola for times indicated.
(J) ChIP-qPCR analysis of phospho-p53 at promoters of indicated genes in wt and *Parp1*$^{-/-}$ mESC treated with 50 nM Tal for 6 h. Shown are mean percentages of input + SEM (n=4 for *Parp1*$^{+/+}$, n=2 for *Parp1*$^{-/-}$ cells).
(K) ChIP-qPCR analysis of H3K4me3 at promoters of indicated genes in wt mESC treated with 5 nM Tal for 4 h. Shown are mean percentages of input + SEM (n=3). Numbers above bars indicate P-values of unpaired, two-tailed t-tests between indicated conditions.

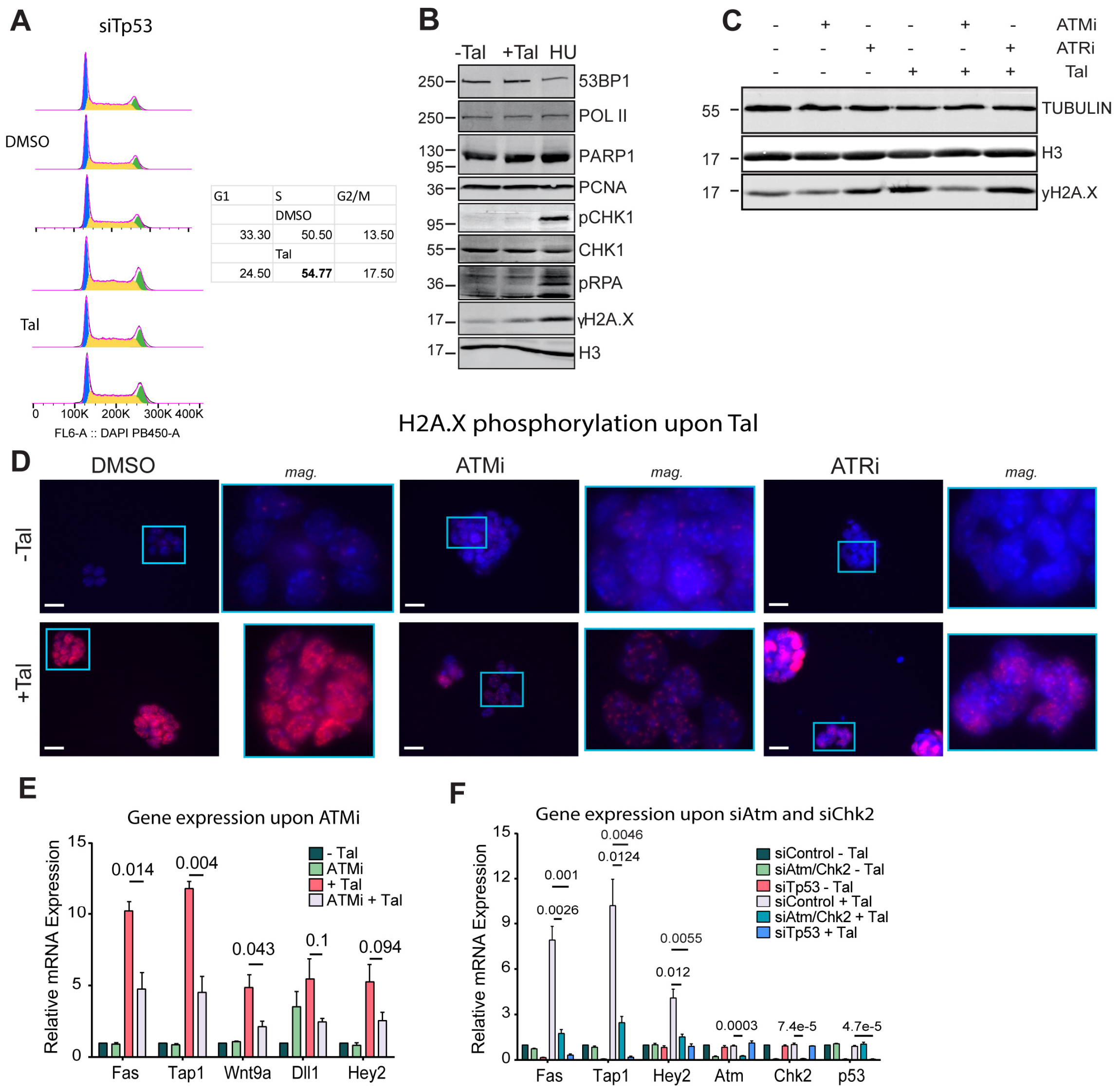

Supplementary Figure 2. Related to Figure 2; ATM-dependent phosphorylation of p53 is the central mediator of the Tal response in naïve mESC

(A) Cell cycle analysis of mESC treated 48 h after transfection with siTrp53. DMSO or 5 nM Tal treatment was added 24 h after transfection.
(B) Immunoblot analysis of wt mESC treated with 5 nM Tal for 4 h or 2 mM hydroxyurea (HU) together with 1 µM ATR inhibitor (AZ20) for 1 hr (n=3).
(C) Immunoblot for γH2A.X in WT mESC treated with 5 nM Tal for 4 h upon ATM (10 µM KU-55933) or ATR inhibition (1 µM AZ20).
(n=3).
(D) Representative images (n=3) show immunofluorescence staining of γH2A.X (red) and DAPI (blue) in wt mESC treated with 5 nM Tal for 16 h upon ATM (10 µM KU-55933) or ATR inhibition (1 µM AZ20). Scale bar: 20 µm. γH2A.X foci are accompanied by diffuse nuclear staining and are visible upon magnification (*mag.*, cyan crop out).
(E) mRNA expression (RT-qPCR) relative to DMSO in WT mESC treated with 5 nM Tal for 6 h upon ATMi (10 µM KU-55933) for 4 h. Data are shown as mean + SD (n=3).

(F) mRNA expression relative to DMSO measured by RT-qPCR analysis in WT mESC treated with 5 nM Tal for 6 h upon Atm or Chk2 KD. Data are shown as mean + SD (n=3). Numbers above bars indicate P-values of unpaired, two-tailed t-tests between indicated conditions.

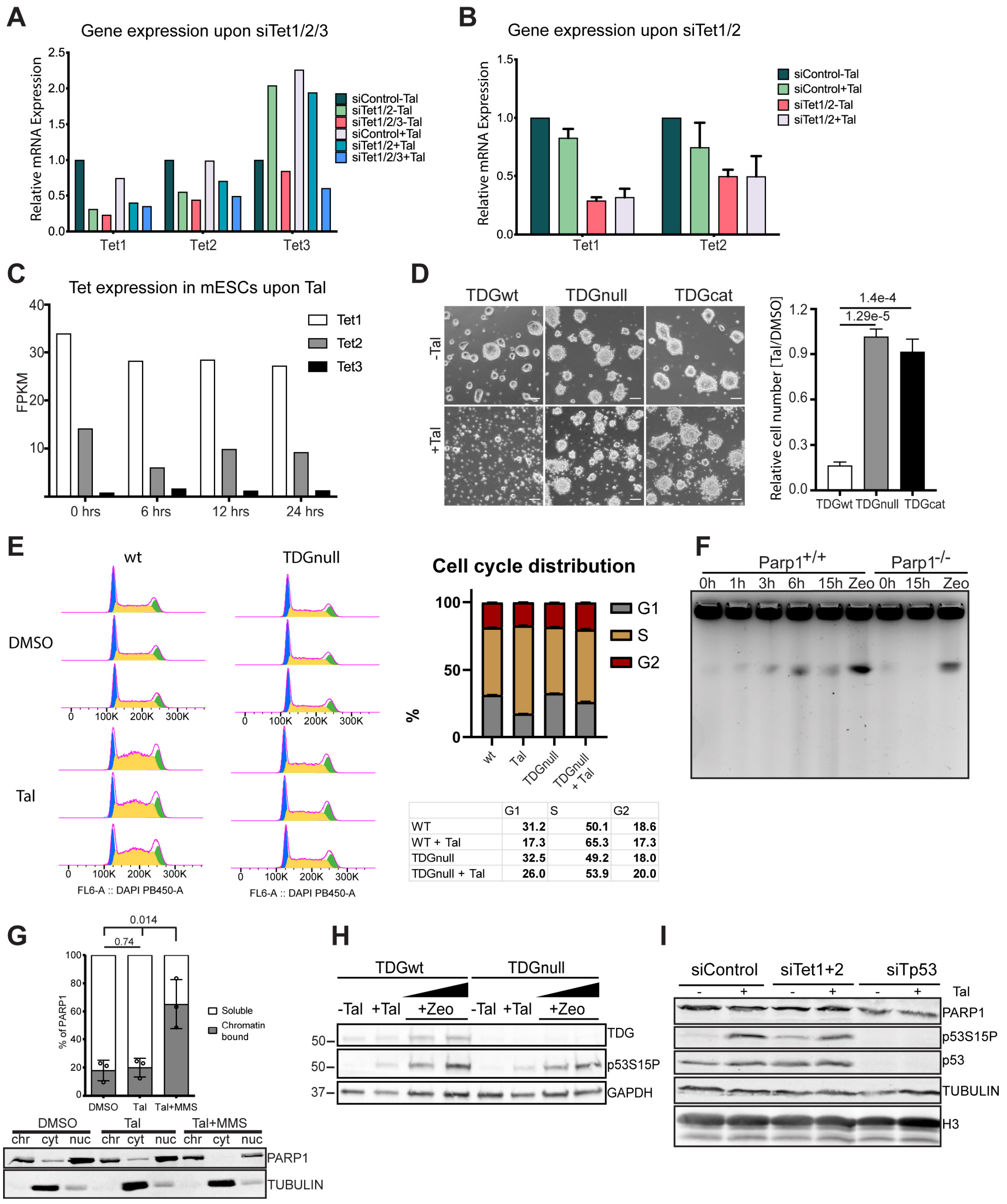

Supplementary Figure 3. Related to Figure 3; TET/TDG-mediated active DNA demethylation facilitates p53-dependent gene expression

(A) RT-qPCR analysis for siRNA efficiency after siTet1/2/3 transfection in wt mESC. Data are shown as mean relative to the control treatment.

(B) RT-qPCR analysis for siRNA efficiency after siTet1/2 transfection in wt mESC Data are shown as mean + SD relative to the control treatment (n=3).

(C) Tet1/2/3 mRNA expression by mRNA-seq in wt mESC treated with 5 nM Tal for indicated time periods.

(D) Left, representative depictions of the Tal sensitivity of TDGwt/null/cat complemented *Tdg*$^{-/-}$

mESC. Cells were treated with 5 nM Tal for 2 days. Scale bar, 40 µm. Right, quantitation of viable cells via Trypan blue staining, data are shown as mean + SD (n=3).

(E) Cell cycle analysis of TDGnull mESC with or without 5 nM Tal treatment for 1 day. For easier comparison, wt data are the same as in Supplementary Fig. 1.

(F) Representative PFGE of wt and *Parp1*$^{-/-}$ mESC treated with 5nM Tal for times indicated. Treatment with 10 ug/ml Zeocin (Zeo) for 24 h served as a positive control for DSB.

(G) left: Representative immunoblot image of fractionated mESC upon 5 nM Tal (and 0.01% MMS) probed for PARP1 and Tubulin. Chr: chromatin bound, cyt: cytoplasmic, nuc: nuclear soluble. right: Quantification of chromatin bound or soluble PARP1. Depicted is the mean +/- SD of n=3. Previously published in Schwarz and Xu et al. 2024 as Supplementary Fig. 4a)

(H) Immunoblot analysis of wt mESC treated with 5 nM Tal for 6 h upon siRNA depletion of TET or p53 (n=3).

(I) Immunoblot analysis of p53 phosphorylation upon 5 nM Tal or 10/20 ug/ml Zeocin (Zeo) in uninduced or induced *Tdg*$^{fl/fl}$ mESC.

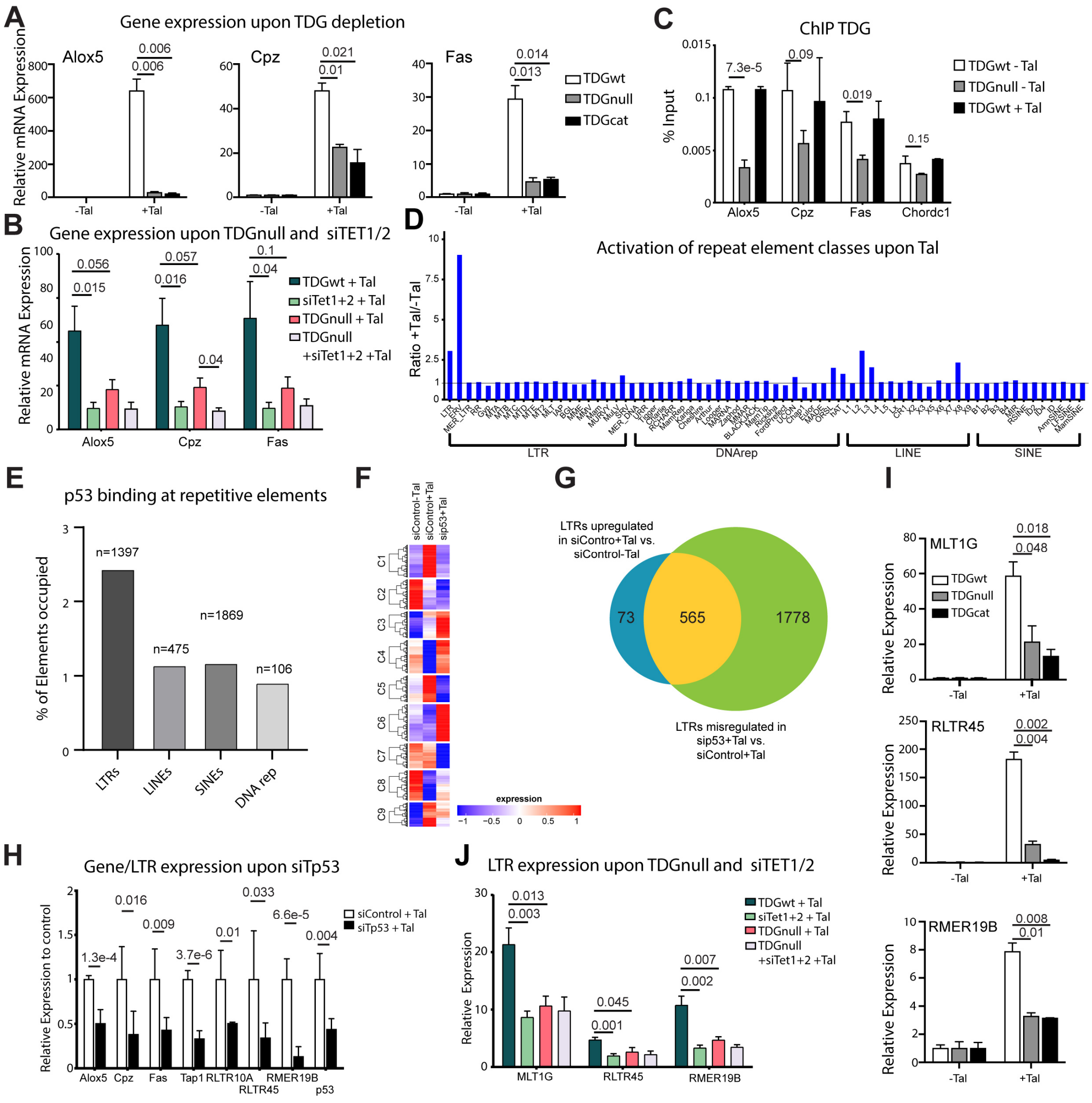

Supplementary Figure 4. Related to Figure 4; Tal induces ERV expression in a p53- and TDG-dependent manner

(A) Relative mRNA expression by RT-qPCR analysis in TDG wt/null/cat-complemented *Tdg*$^{-/-}$ mESC treated with 5 nM Tal for 24 h. Shown are means + SD (n=2).
(B) Relative mRNA expression by RT-qPCR analysis in in *Tdg*$^{fl/fl}$ mESC treated with 5 nM Tal for 24 h with or without additional siRNA-mediated depletion of TET1/2. Relative data to DMSO treated cells are shown as mean + SD (n=2).
(C) ChIP-qPCR analysis of TDG in *Tdg*$^{fl/fl}$ mESC treated with 5 nM Tal for 24 h. Shown are means + SD (n=3 for uninduced cells, n=2 for induced cells). A non-targeted region ~120kb upstream of *Chordc1* was used as a negative control.
(D) Tal-induced repeat element expression from total RNA-seq in mESC treated with 5 nM Tal for 6 h vs. DMSO treated mESC.
(E) Proportion of reliably detected repeat elements (min 10 counts, totalRNA-seq) displaying ChIP-seq evidence for DNA stress-induced p53 occupancy (Li et al 2012). n = absolute number of detected repeats with p53 binding.

(F) Heat map showing significantly differentially expressed ERVs in wt or siTP53 treated mESC with or without 5 nM Tal treatment for 6 h.
(G) Overlap between Tal activated LTRs (blue circle) and LTRs whose activation by Tal was reduced upon p53 depletion (green circle).
(H) Relative RNA expression (RT-qPCR) in *Tdg*$^{fl/fl}$ mESC treated with 5 nM Tal for 24 h upon siTp53. Shown are means of relative gene expression (+Tal/-Tal) + SEM (n=5).
(I) Relative RNA expression of LTRs (RT-qPCR) in TDGwt/null/cat-complemented *Tdg*$^{-/-}$ mESC treated with 5 nM Tal for 24 h. Shown are means + SD (n=2).
(J) Relative RNA expression of LTRs (RT-qPCR) in *Tdg*$^{fl/fl}$ mESC treated with 5 nM Tal for 24 h with or without additional treatment with siTET1+2. Shown are means + SD (n=2). Numbers above bars indicate P-values of unpaired, two-tailed t-tests between indicated conditions.

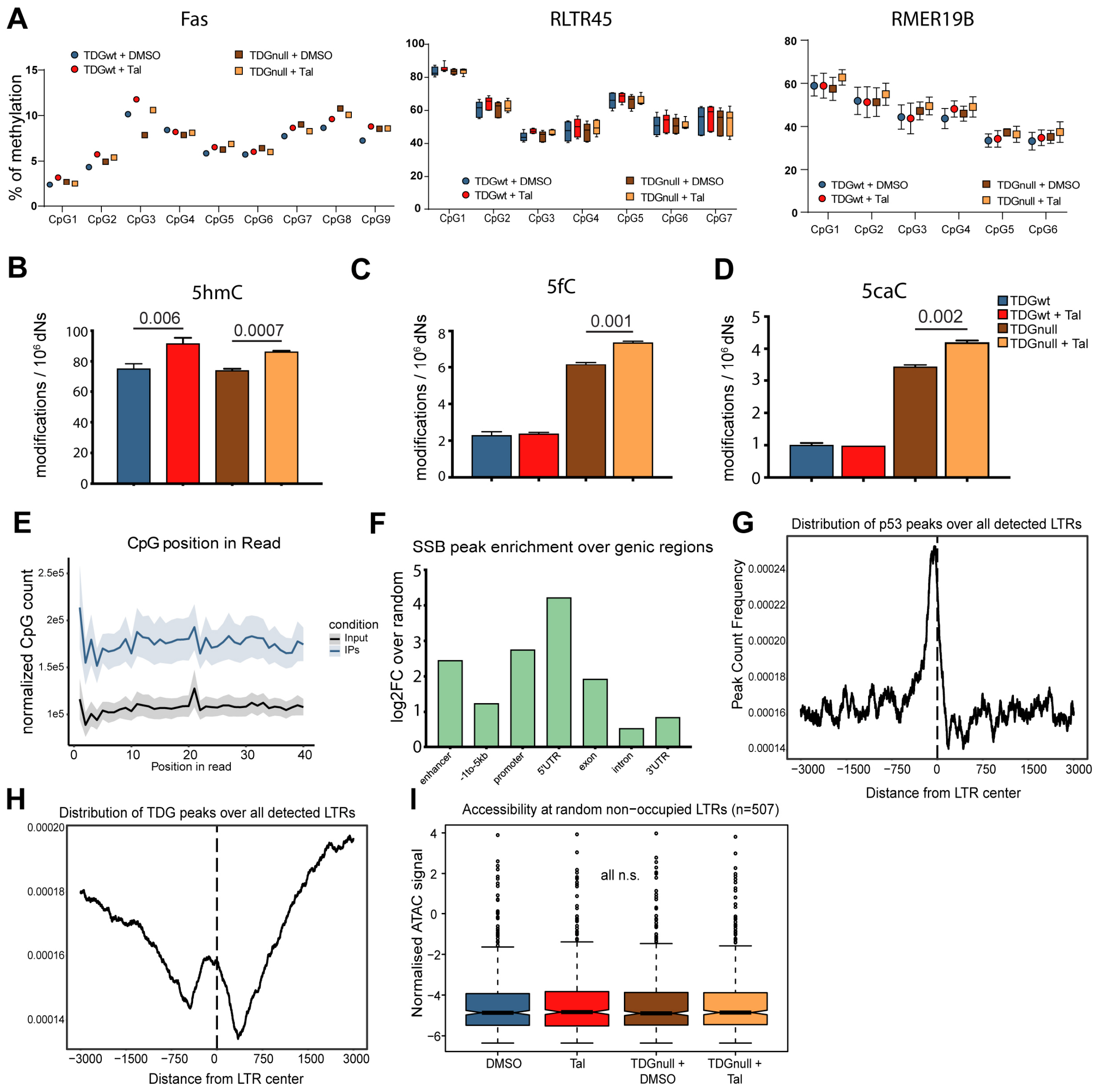

Supplementary Figure 5. Related to Figure 5 Tal treatment induces SSB at ERVs in a TDG dependent manner.

(A) 5mC analysis by bisulfite-assisted pyrosequencing in *Tdg*$^{fl/fl}$ mESC treated with 5 nM Tal for 24 h. Shown are methylation levels of individual CpGs in following regions: Fas: TSS +26/+78 bp (n=1), RLTR45: TTS -93/+6 bp (n=6), RMER19B: TTS+109/+187 bp (n=3)

(B-D) Mass spectrometry analysis of 5hmC, 5fC and 5caC in *Tdg*$^{fl/fl}$ mESC treated with 5 nM Tal for 24 h. Shown are means + SD (n=3).

(E) Normalized CpG distribution in IP- and input reads from SSB-seq. Shadings indicate confidence intervals over all conditions and replicates.

(F) Distribution of SSB-enriched regions at genic regions indicated, shown as log2-fold enrichment over the same number of randomly sampled genomic regions with equal length.

(G) Density of p53 from published ChIP-seq data peaks (Li et al. 2012) over detected LTRs.

(H) Density of TDG-enriched genomic regions (Neri et al. 2015) over detected LTRs.

(I) Normalized ATAC signal at randomly sampled LTRs with neither p53 or TDG occupation (n=506).

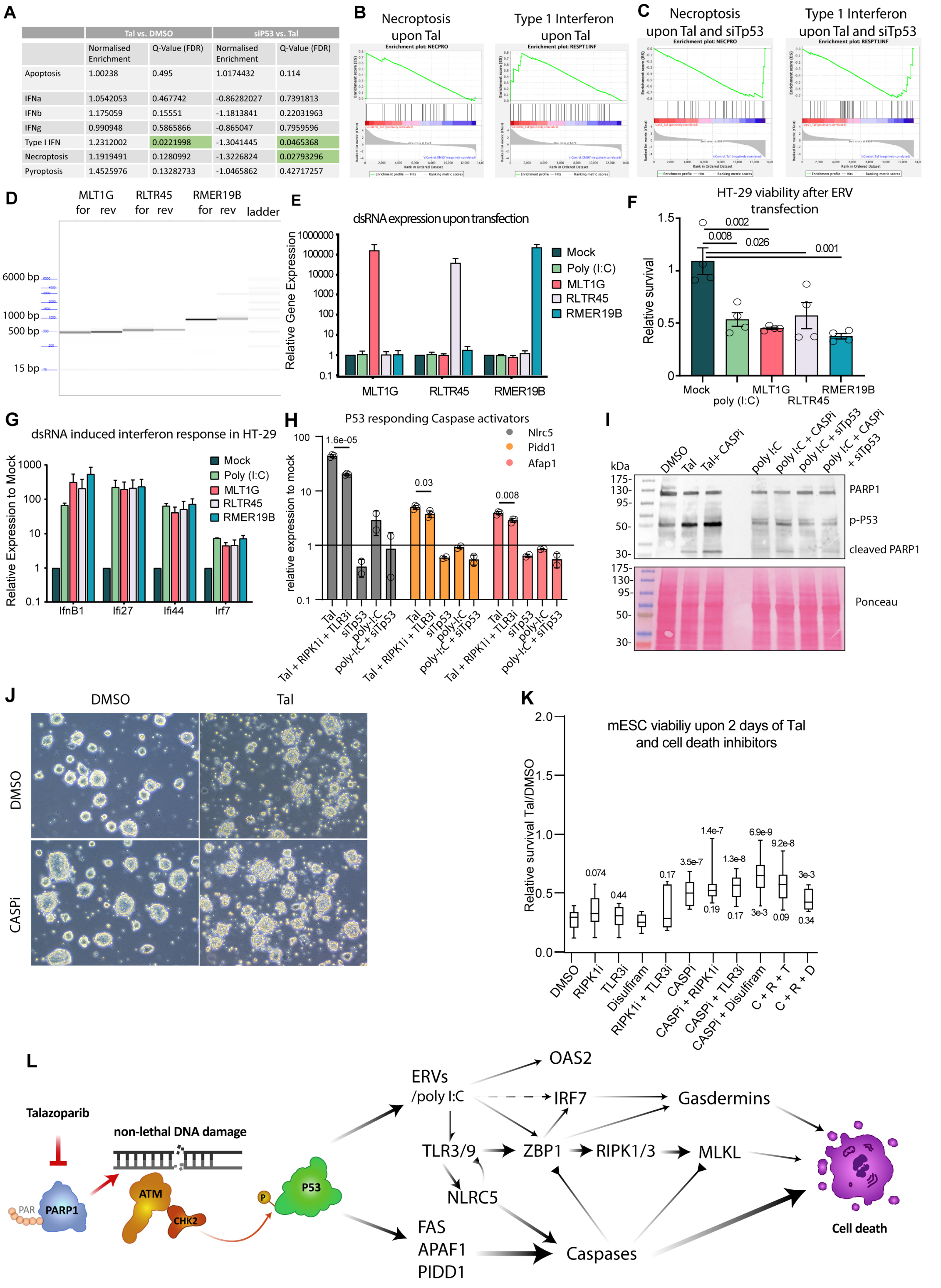

Supplementary Figure 6. Related to Figure 6; Tal-mediated ERV de-repression causes a necroptosis and interferon-like response

(A) Enrichment scores and adjusted P-values of GSEA of indicated pathways.

(B) GSEA of Necroptosis (left) and Type I Interferon response (right) genes of wt mESC in response to

5 nM Tal for 6 h.

(C) GSEA of Necroptosis (left) and Type I Interferon response (right) genes in siTp53-treated mESC in response to 5 nM Tal for 6 h.

(D) Fragment analysis of forward and reverse strand of *in vitro* transcribed RNA.

(E) RT-qPCR analysis of transfected dsRNAs in wt mESC. Shown as mean + SD (n=2).

(F) Relative number of trypan blue negative HT-29 cells transfected with *in vitro*-transcribed ERV dsRNA as indicated, 2 days after transfection (1 ug/ml medium). Shown are means with SEM (n=4).

(G) Relative mRNA expression (RT-qPCR) of known interferon-responsive genes in HT-29 cells after treatment as in **f**. Error bars represent the SD of n=2.

(H) Expression (RT-qPCR) of known Caspase activators upon Tal and indicated inhibitor or siTp53 treatment.

(I) Immunodetection of phosphorylated p53(p-P53) and PARP1 in mESC after 1 day of treatment with Tal (5 nM), CASPi (25 µM), poly-I:C (1 µg/ml) and/or siTp53.

(J) Representative images of mESC with indicated treatments.

(K) WST-assay displaying metabolic activity of mESC treated with Tal (5 nM) for 2 days after 1 day of pre-incubation and following co-incubation for 2 days with: RIPK1i (Necrostatin-1, 60 µM), TLR3i (CU-CPT-4a, 7.5 µM), CASPi (Z-VAD-FMK, 25 µM), Disulfiram (200 nM). Number above boxes indicate P-value of unpaired t-test of the condition vs. DMSO. Numbers below indicate unpaired t-test of the condition vs. CASPi. no number indicates P-value > 0.5. n(C+N+D) = 4, n(others): 8-12. Data is the continuation of data in Fig. 6f.

(L) Working model with more detailed schematic of investigated cell death factors at play.
